# Transmembrane Domain Lengths Serve as Signatures of Organismal Complexity and Viral Transport Mechanisms

**DOI:** 10.1038/srep22352

**Published:** 2016-03-01

**Authors:** Snigdha Singh, Aditya Mittal

**Affiliations:** 1 Kusuma School of Biological Sciences, Indian Institute of Technology Delhi, Hauz Khas, New Delhi 110016, India.

## Abstract

It is known that membrane proteins are important in various secretory pathways, with a possible role of their transmembrane domains (TMDs) as sorting determinant factors. One key aspect of TMDs associated with various “checkposts” (i.e. organelles) of intracellular trafficking is their length. To explore possible linkages in organisms with varying “complexity” and differences in TMD lengths of membrane proteins associated with different organelles (such as Endoplasmic Reticulum, Golgi, Endosomes, Nucleus, Plasma Membrane), we analyzed ~70000 membrane protein sequences in over 300 genomes of fungi, plants, non-mammalian vertebrates and mammals. We report that as we move from simpler to complex organisms, variation in organellar TMD lengths decreases, especially compared to their respective plasma membranes, with increasing organismal complexity. This suggests an evolutionary pressure in modulating length of TMDs of membrane proteins with increasing complexity of communication between sub-cellular compartments. We also report functional applications of our findings by discovering remarkable distinctions in TMD lengths of membrane proteins associated with different intracellular transport pathways. Finally, we show that TMD lengths extracted from viral proteins can serve as somewhat weak indicators of viral replication sites in plant cells but very strong indicators of different entry pathways employed by animal viruses.

Proper subcellular localization of membrane proteins into different organelles/membrane-environments is one of the keys to survival and propagation of living cells. Thus, identification of subcellular localization of novel membrane proteins in eukaryotic cells is an important step towards understanding their role in the life of a cell. Several years ago, transmembrane domains (TMDs) of integral membrane proteins were shown to contain factors that control their sorting in secretory pathways^1^,^2^,^3^. While there is general consensus on cytosolic sorting signals being responsible for determining intracellular trafficking pathways and subsequent subcellular location of membrane proteins, the role of their own TMDs in sorting of these membrane proteins has started emerging prominently^4^. In fact, TMD-dependent sorting has been proposed to be more significant than cytosolic sorting signals for membrane proteins^5^. In this regard, it is interesting to note that around the same time as demonstration of role of TMDs in the secretory pathway, it was reported that membrane proteins with TMDs shorter by (on an average) five amino acids than TMDs of plasma membrane proteins were retained in the cisternae of the Golgi complex^6^. Additional studies in yeast showed that proteins with longer TMDs were targeted to the plasma membrane while those with shorter TMDs were targeted to vacuole^7^,^8^,^9^. More recently, some studies reported that proteins with very short TMDs are specifically targeted for endocytosis by clathrin-coated vesicles^10^. Interestingly, an elegant proteomic analysis carried out by Munro and colleagues led to strong insights on the difference in TMD lengths of membrane proteins associated with different organelles along secretory pathways in fungal and vertebrate cells^11^. The conceptual simplicity in lengths of TMDs of intracellular membrane proteins serving as signatures for their respective intracellular locations/organelles in fungi and vertebrates is very appealing. If this finding can be generalized/extrapolated to all living systems, it would be very promising towards obtaining mechanistic insights into intracellular trafficking using proteomics—especially due to relatively straightforward algorithms for detection and prediction of TMDs in known protein sequences, a very large majority of which are yet to be structurally resolved. Thus, in this work, we carried out a comprehensive TMD length analysis on ~70000 membrane protein sequences corresponding to 301 genomes of fungi, plants, non-mammalian vertebrates and mammals. The first remarkable result we report in this work, while confirming the original findings of Sharpe *et al.*^11^ for fungi and vertebrates, is a decrease in variation of TMD lengths of membrane proteins with increasing organismal complexity (i.e. difference in TMD lengths of organellar membrane proteins and plasma membrane proteins decreases as we move from “simpler” to “complex” organisms). This result provides a profound insight towards increased sharing/exchange of intracellular and plasma membrane components with increasing organismal complexity over time.

The fact that we were able to confirm and substantially generalize that TMD lengths indeed serve as signatures for subcellular locations in different eukaryotic systems, we decided to further explore the scope of our findings from an extremely important applicational perspective. Viruses in both animals and plants, regardless of presence^12^,^13^,^14^,^15^,^16^ or absence of a membrane envelope, rely heavily on intracellular trafficking and sorting^17^ mechanisms of their host cells, including viral replication associated with host intracellular membranes^18^. Mechanistic insights into viral entry and exit mechanisms provide strong avenues of controlling their pathogenic activity. Therefore, we analyzed protein sequences from 34 different viruses (19 infecting animal cells and 15 infecting plant cells) and extracted their TMD lengths by using the methodology developed in this work. The key hypothesis to test was that TMD lengths of viral proteins may provide signatures of subcellular locations (i.e. membrane/organellar) of internalization- or secretory- or replication- pathways associated with their life cycles in respective host cells. We specifically analyzed experimentally determined viral protein sequences that are known to play a key role in their entry (in case of animal viruses), and replication (in case of plant viruses), into their respective hosts – thus serving as strong experimentally determined controls for our analyses. With this approach, we report that TMD lengths of viral proteins do indeed serve as signatures for important host cell “checkposts” (i.e. membrane/organellar host cell locations) involved in life cycles of both animal and plant viruses. To our knowledge, this is a first-of-a-kind study, especially involving plant viruses, along with several animal viruses, showing that solely TMD lengths (independent of actual primary sequences) of viral proteins serve as signatures of subcellular locations important in viral life cycles. This work opens up a very promising avenue for designing experiments aimed at interfering with viral transport mechanisms for both animal and plant viruses using a relatively straightforward, yet rigorous and somewhat computationally economical, analytical approach.

## Results

### Evaluation of TMDs of single span membrane proteins localized to their associated compartments i.e. Golgi, Endoplasmic reticulum, Nucleus, TGN/endo and Plasma Membrane from different organisms (Fungi, Plants, Non-Mammalian Vertebrates and Mammals)

**Figure 1:**
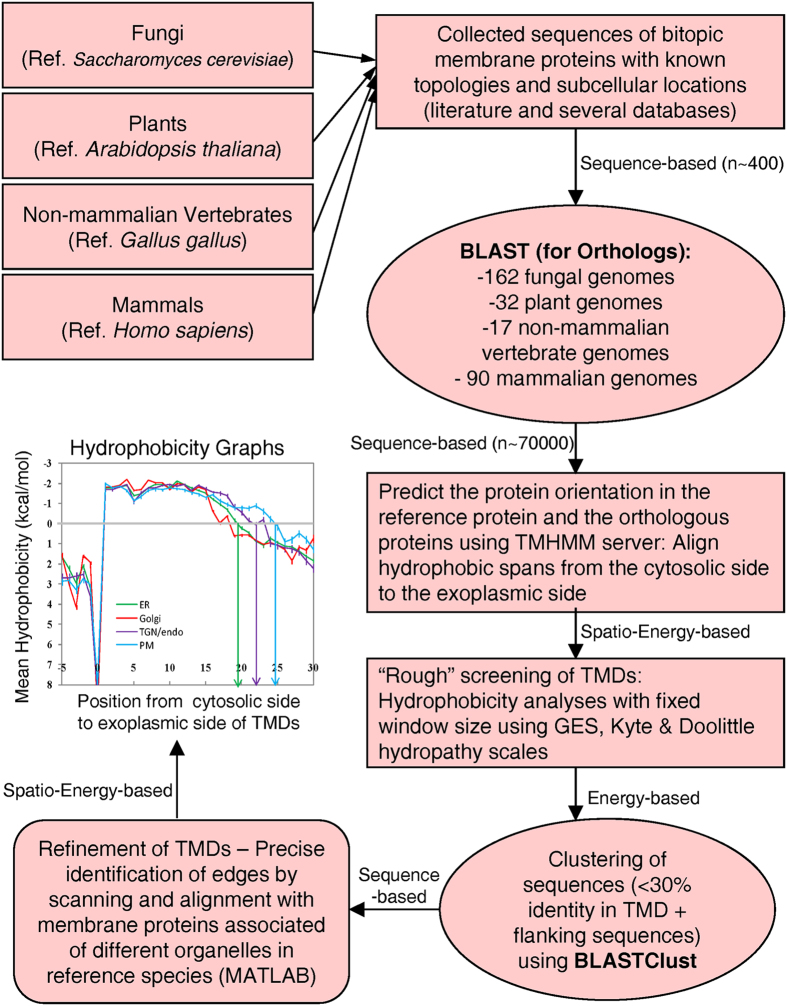
A Sequence-Spatio-Energy-based methodology for trans-membrane domain (TMD) analysis. Trans-membrane proteins of known topology and location were identified from literature and databases for different organisms (fungi, plants, non-mammalian vertebrates and mammals). Reference species for each organism is shown in parentheses below the organism name. Orthologous proteins were identified using BLAST searches. Subsequent to prediction of protein orientation, a “rough” screening for TMDs and assignment of up to eight flanking residues on both sides of TMDs was done in all sequences. Then, BLASTClust was used to remove sequence redundancy by collecting protein sequences which were not very similar (<30% identity) to respective reference protein, and with each other, in order to cover a wide range of protein sequences. Finally, refinement of TMDs was done in which all the protein sequences from a dataset (for example from an organelle of an organism) were aligned at the positions where a sharp change in hydrophobicity (hydopathy) occurred. The cytosolic end of hydrophobic region was assumed as position one and the hydrophobic spans were aligned from the cytosolic side to the exoplasmic side. Arrows connecting each step are also marked with number of sequences involved in that step, and a description of whether the step involved sequence-based, spatially-based, and energy-based or a combination of any of these analyses.

To compare the TMDs from different organelles we extracted bitopic proteins (i.e. with only one TM helix) from all membrane proteins of eukaryotic genomes. Bitopic proteins include many important families of receptors many of which are fruitful targets for biopharmaceuticals and also mutations in bitopic protein are frequent cause of various human diseases such as cancer or developmental disorder. For our analysis we collected reference proteins from the best characterized eukaryotic genomes, *Saccharomyces cerevisiae* (Supplementary Table S1)*, Arabidopsis thaliana* (Supplementary Table S2)*, Gallus gallus* (Supplementary Table S3) and *Homo sapiens* (Supplementary Table S4). To expand our dataset we used BLAST to include orthologs in our dataset. Here, it needs to be emphasized that if orthologous proteins were very much similar to reference proteins and among themselves also, then our analyses would be biased and probably less meaningful. Therefore, to avoid such bias in analyzing membrane protein sequences, we used BLASTClust to cluster these proteins on the basis of their TMDs and flanking sequences, finally selecting only those proteins which were not very similar (less than 30% identity) to each other from each organelle set (Fig. 1). For first screening of identifying membrane proteins, hydrophobicity (also called hydropathy) profiles with an average window size of 18 were scanned – the actual TMD identification was done after a computationally intensive analysis refining the hydropathy profile data in an unbiased manner by utilizing a scanning and alignment algorithm developed (see Methodology for details). The accuracy of the developed computational method was rigorously tested by repeating exact results obtained by Sharpe *et al.*^11^.

### Mean Lengths of TMDs

It is important to note that the hydrophobicity graph shown in Fig. 1 represents the averages of hydrophobicity profiles of all the helices—with this calculation the hydrophilic-hydrophobic crossing point (e.g. at 0 kcal/mol) reflects the dominant value rather than the outliers. Specifically, for example, the lengths of TMDs obtained by counting from 0 to the tip of the arrows shown in Fig. 1 represent the most common values of the graphs (the modes), rather than the means. However, for meaningful interpretation of comparative TMD lengths it is important to analyze and compare the mean, rather than only the mode, since it is imperative to analyse membrane proteins in each organelle class as a whole, rather than the most dominant sub-population of membrane proteins. Thus, the mean hydrophobic lengths of TMDs, for each of the organelles in each of the organisms analyzed, are shown in Table 1.

**Figure 2:**
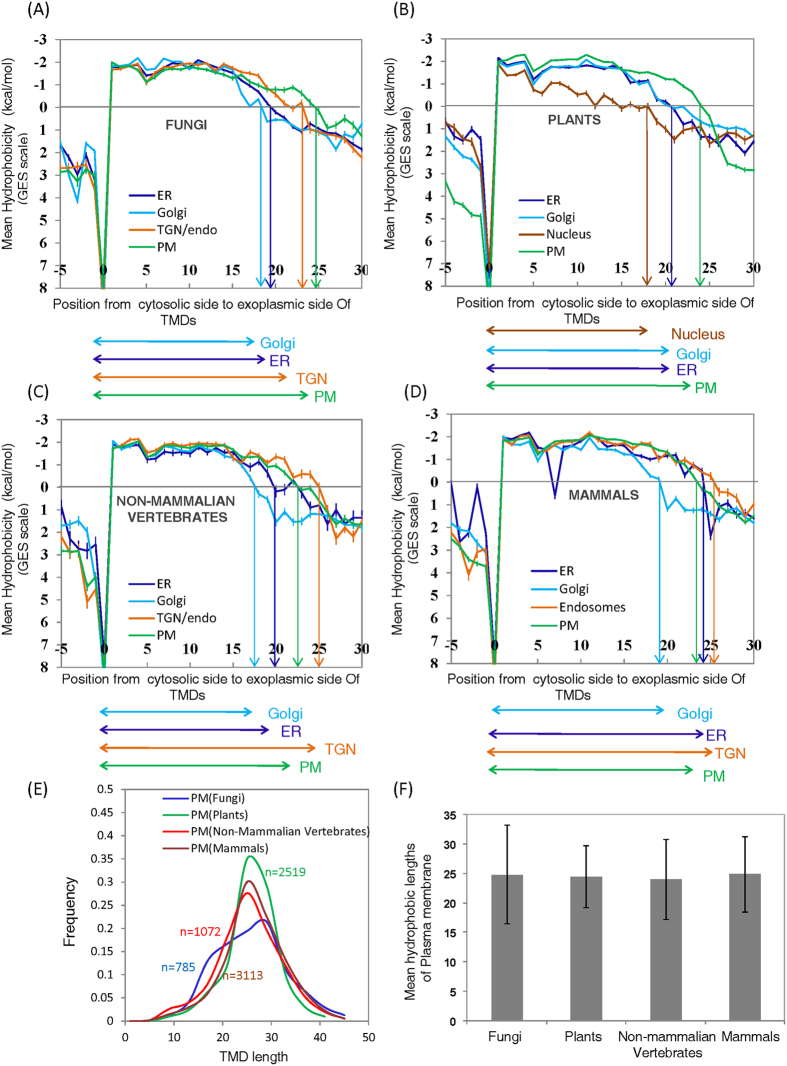
Differences in hydrophobicity profiles of TMDs of all the organelles in fungi, plants, non-mammalian vertebrates and mammals (GES Scale). (**A**) Analysis of hydrophobicity profiles of TMDs of proteins from different organelles (Golgi, Endoplasmic Reticulum—ER, TGN/endo and Plasma Membrane—PM) along the secretory pathway in fungi. This figure is directly inspired by the work of Sharpe *et al.*^11^ – it was essential to reproduce their earlier results with our extended data set. Therefore, axes and color coding (for organelles) are retained – the figure serves as strong positive (computational) control for the methodology developed in this work. (**B**) Analysis of hydrophobicity profiles of TMDs of proteins from different organelles (Golgi, ER, Nucleus and PM) in plants along the secretory pathway. Clearly, plasma membrane proteins in plants also have TMDs longer than proteins of all other organelles. (**C**) Analysis of hydrophobicity profiles of TMDs from different organelles (Golgi, ER, TGN/endo and PM) along the secretory pathway in non-mammalian vertebrates. (**D**) Analysis of hydrophobicity profiles of TMDs of proteins from different organelles (Golgi, ER, TGN/endo and PM) in mammals along the secretory pathway. (**E**) Distribution plots of TMD lengths of plasma membrane proteins from different organisms (fungi, plants, non-mammalian vertebrates and mammals). (**F**) Bar chart showing mean hydrophobic lengths of TMDs of plasma membrane proteins in different organisms.

Recently Sharpe *et al.*^11^ showed that, on an average, the hydrophobic length for plasma membrane TMDs is larger than those of ER and Golgi in both fungi and vertebrates. While the data of Sharpe *et al.*^11^ is a subset of the data analyzed by us, it is not only interesting that our larger data set on fungi confirms their findings, it is also remarkable that those findings still hold true for organellar TMDs vs. plasma membrane TMDs when data from plants is analyzed (Fig. 2A,B). Even more remarkable is the fact when we analyze much larger data sets for vertebrates, and parse them into non-mammalian and mammalian, Golgi TMDs are found to be shorter than plasma membrane TMDs (Fig. 2C,D). Thus, while confirming earlier findings, our analysis substantially generalizes the conclusions reached by Sharpe *et al.*^11^ in terms of variety of organisms. An important aspect with respect to the possible common cellular origins of all organisms, regardless of the level of their complexity at the whole organism level, was also confirmed by us – Fig. 2E shows the distribution plots of lengths of plasma membrane TMDs of different organisms. Figure 2F expresses the same results (i.e. those of Fig. 2E) in terms of mean ± std as a bar graph. It can be clearly seen from Fig. 2E,F that plasma membrane TMDs for all variety of organisms analyzed by us are similar. This is a clear indication of a common cellular evolution with respect to the cellular boundaries regardless of organismal complexity.

### Differences in TMD lengths among different organelles in fungi, plants, non-mammalian vertebrates and mammals

**Figure 3:**
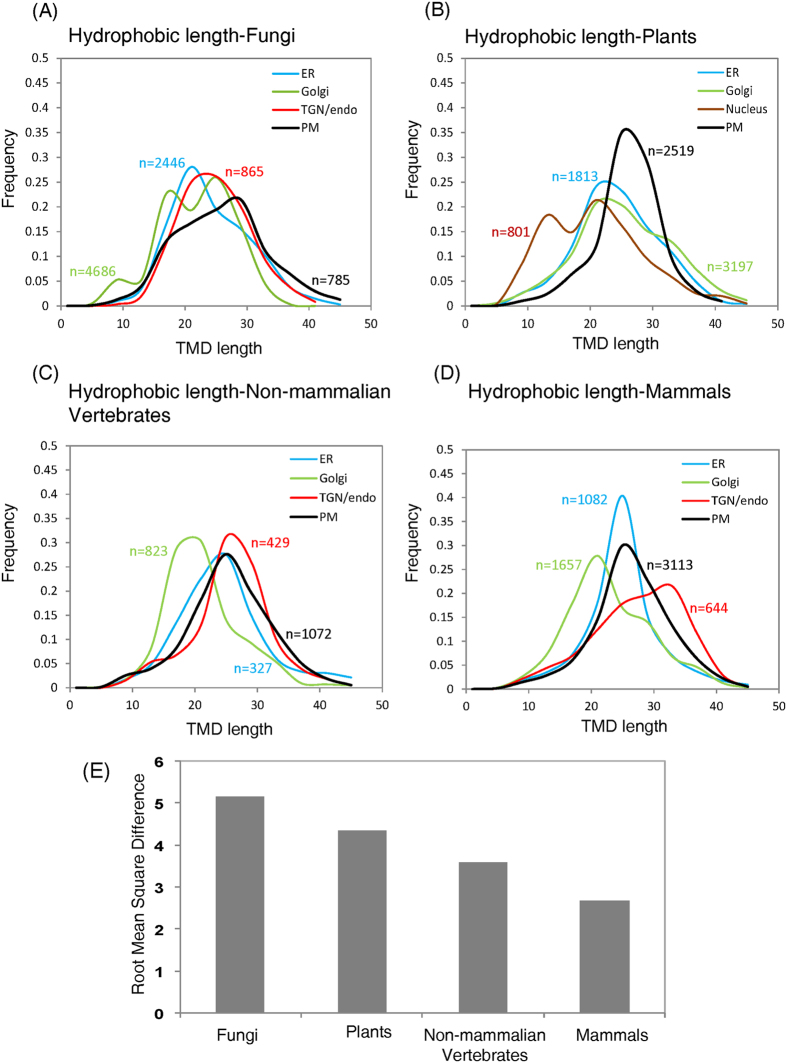
Distribution plots of TMD lengths of proteins in (**A**) fungi, (**B**) plants, (**C**) non-mammalian vertebrates, and, (**D**) mammals. Organelles were the same as in Fig. 2. X-axes represent TMD lengths and Y-axes represent frequencies, with n = number of transmembrane protein sequences associated with each organelle in each of the organisms, (**E**) Bar chart showing variation in the differences of TMD lengths of proteins among different organelles with respect to PM in different organisms. The root mean square of difference for an organism was calculated by taking the square root of the sum of squares of the difference between TMD length of each protein associated with each organelle and corresponding PM of each species in that organism.

As mentioned earlier, our findings show that Golgi TMDs have lesser TMD length than plasma membrane TMDs (Fig. 2), confirming earlier conclusions^11^, but with a much larger dataset using the GES hydrophobicity scale. To further ensure analytical rigor of the results, and, that these findings are not “hydrophobicity-scale-dependent”, we confirmed that the same conclusions are reached from hydrophobicity graphs plotted on the basis of Kyte-Doolittle scale (Supplementary Fig. S1). Below we discuss the differences in TMD lengths, among different organelles for different organisms, with distribution profiles shown in Fig. 3A–D.

#### Fungi

We observed that the length of TMDs of plasma membrane is highest compared to different organelles and not just the Golgi (Supplementary Fig. S2A). Analysis of amino acid composition of TMDs from cytosolic to exoplasmic side for different organelles in fungi indicate that the regions abundant in hydrophobic residues are larger in TGN/endo and plasma membrane proteins compared to ER and Golgi proteins—showing a difference in TMD length (Supplementary Figs S3A and S4). Additionally, we found that hydrophobic residues occupy smaller regions along the TMDs in Golgi than in plasma membrane (Supplementary Fig. S5). While the distribution plots of TMD lengths of all organelles in fungi, shown in Fig. 3A, clearly indicate distinct (and shorter) TMD lengths compared to plasma membranes (black distribution in Fig. 3A), rigorous statistical analyses shown in Table 2, i.e. small “p” values in t-tests and Kullback-Leibler Divergence Measure (KLDM) values, confirm that the differences in the TMD lengths of all the organelles are highly significant, especially w.r.t. plasma membrane, and from each other.

#### Plants

We observed from hydrophobicity graphs of plants that length of TMDs of Golgi and ER were almost same but length of TMDs of Mitochondria, Nucleus, Peroxisomes, Chloroplasts and Plasma membrane were different (Fig. 2B and Supplementary Fig. S6). Distributions of TMD lengths in plants clearly indicated longer TMD lengths for plasma membranes, as shown in Fig. 3B (corresponding mean ± std is shown as a bar graph in Supplementary Fig. S2A). Analysis of amino acid composition of TMDs from different organelles in plants indicates that the regions abundant in hydrophobic residues are almost similar in ER and Golgi membrane and the regions abundant in hydrophobic residues are minimum in nuclear membrane proteins and maximum in plasma membrane proteins (Supplementary Figs S3B and S4). Additionally we observed in our abundance graphs that area of nuclear membrane TMDs enriched in hydrophobic residues is smaller as compared to TMDs of plasma membrane (Supplementary Fig. S5). As done earlier, rigorous statistical analyses shown in Table 2, i.e. small “p” values in t-tests and Kullback-Leibler Divergence Measure (KLDM) values, confirm that the differences in the TMD lengths of all the organelles are highly significant, especially w.r.t. plasma membrane, and from each other (except ER and Golgi).

#### Non-mammalian vertebrates

In non-mammalian vertebrates, we found that there is a significant difference in TMD lengths of ER and Golgi, but the difference between TGN/endo and plasma membranes was not significant (Fig. 2C, Table 2). Distribution plots of TMD lengths of all the organelles show relatively less distinct profiles (Fig. 3C) and the differences in the TMD lengths of all the organelles are much lower as compared to those observed in fungi and plants (Table 2). Analysis of amino acid composition of TMDs from different organelles in non-mammalian vertebrates indicate that the regions abundant in hydrophobic residues are somewhat similar in TGN/endo and in plasma membrane proteins, and in ER and Golgi proteins the regions abundant in hydrophobic residues are smaller than TGN/endo and plasma membrane (Supplementary Figs S3C and S4). In abundance graphs of non-mammalian vertebrates, we observed that hydrophobic residues occupy almost same area along the TMDs in plasma membrane than in TGN/endo membrane proteins (Supplementary Fig. S5). Thus, in non-mammalian vertebrates, TMD lengths are similar for TGN/endo and plasma membranes, and these have higher TMD lengths compared to other organelles. Interestingly, the overall differences between the plasma membrane TMD lengths and other organelles are much lower compared to fungi and plants.

#### Mammals

Mean hydrophobic lengths of TMDs of endoplasmic reticulum, endosomes and plasma membrane were found to be similar in mammals, indicating that the thickness of bilayers of these organelles is similar, in contrast to other organelles of fungi, plants and non-mammalian vertebrates. However there is still a significant difference (even though less significant than other organisms) among these organelles (Tables 1 and 2). This can also be seen from the distribution plots of TMD lengths of all the organelles (Fig. 3D). Analysis of amino acid compositions of TMDs from different organelles at their different position i.e. from cytosolic side to exoplasmic side in mammals indicates that the regions abundant in hydrophobic residues are almost similar in ER, endosomes and plasma membrane but smaller for Golgi proteins (Supplementary Figs S3D, S4 and S5). Thus, in mammals, as observed in non-mammalian vertebrates, while TMD lengths are similar for TGN/endo and plasma membranes, and these have higher TMD lengths compared to other organelles, the overall differences between the plasma membrane TMD lengths and other organelles are interestingly much lower compared to fungi, plants and non-mammalian vertebrates.

#### Summarizing

The overall differences in TMD lengths of different organelles in fungi, plants, non-mammalian vertebrates and mammals with their respective plasma membranes reveals a very remarkable result, based on GES hydrophobicity scale, shown in Fig. 3E (here it is important to note supplementary Fig. S1F, based on Kyte and Doolittle hydrophobicity scale, confirms that these findings are hydrophobicity scale independent). As we move towards organisms perceived to be more complex (i.e. in terms of organismal complexity fungi < plants < non-mammalian vertebrates < mammals), the overall statistical differences of organelles from respective plasma membranes decreases. Thus, our results suggest that there is gradual decrease in the difference of TMD length, and hence bilayer thickness, among cellular organelles and plasma membrane when we move from “simpler” (or “lower”) organisms to more “complex” (or “higher”) organisms. The variation in hydrophobic length of TMDs of membrane proteins for different organelles, especially when compared with that of their respective plasma membranes, decreases with increase in cellular dynamics and increased organismal complexity. These results are strongly supported by earlier findings that increased intracellular dynamics are one of the key features of higher (more complex) organisms^5^. Higher cell dynamics imply more exchange and/or continuity of material exchange, thereby reducing differences in bilayer thickness of subcellular components – a finding remarkably well extracted by lengths of TMDs of membrane proteins analysed in this work. We address some very exciting aspects of these startling findings in the discussion section.

### Functional application of TMD lengths serving as signatures of organismal complexity towards cellular transport mechanisms

**Figure 4:**
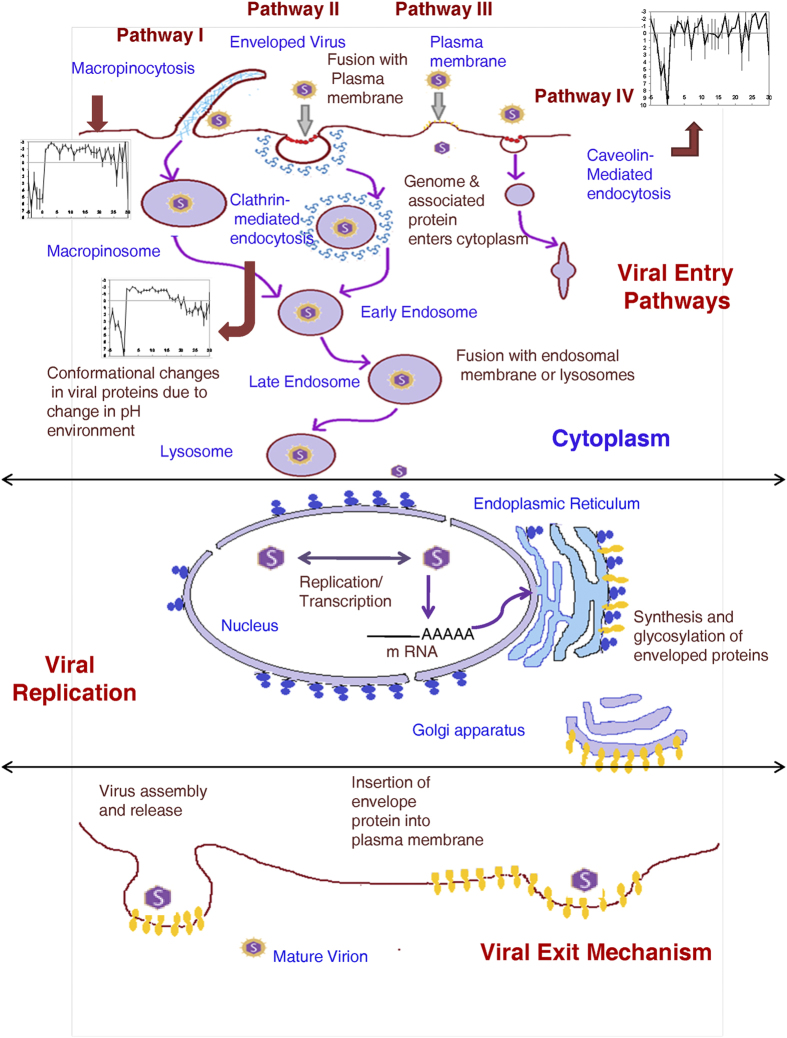
Different entry pathways followed by animal viruses along with virus assembly and exit mechanism. A schematic showing different modes of entry of enveloped animal viruses into host cells. Some viruses enter directly through plasma membrane (Pathway III) but most of the viruses penetrate through endocytic machinery. Pathways I, II and IV represent viral entry into host cells through macropinocytosis, clathrin-mediated endocytosis and caveolin-mediated endocytosis respectively.

Encouraged by our findings on TMD lengths showing organelle specific dependence at a cellular level in different organisms, we decided to explore applicational perspectives of our results. All viruses, regardless of whether they are enveloped (i.e. contain their own membrane bilayer) or non-enveloped, employ intracellular transport mechanisms of their host cells for initiating and propagating infections. These transport mechanisms involve intricate associations and interplay of members of viral proteomes with membranes of different organelles, their by assisting virions in entry into-, and exit from-, host cells. For example, Fig. 4 shows how many animal viruses are known to employ several modes of entry into their host cells – mainly through clathrin-mediated endocytosis, but some also via macropinocytosis, caveolin-mediated endocytosis, plasma membranes and through some other pathways, along with their exit mechanisms. Therefore, we formulated a straightforward question – Given a viral proteome, is it possible to scan for putative hydrophobic segments representing specific TMD lengths to extract information on specific organellar association of the virus during its journey into or out of a host cell ? To answer the above question, first we had to confirm whether host cell proteins themselves, which are involved in intracellular sorting pathways (e.g. clathrin-mediated – and caveolin-mediated – endocytosis, macropinocytosis), have TMD lengths serving as signatures of intracellular sorting. While a regular feature in cell biology textbooks is the important role of cytosolic endocytic signals in endocytosis of membrane proteins, it is emerging that in the absence of cytosolic sorting signals TMDs may act as sorting determining factors during endocytosis. In fact, a few earlier reports encouraged us to hypothesize that TMD lengths can serve as signatures of intracellular sorting^19^,^20^,^21^,^22^. To test this hypothesis, we plotted hydrophobicity graphs of clathrin coat assembly proteins^10^,^23^, along with proteins involved in macropinocytosis^24^,^25^,^26^ and caveolin-mediated^27^ endocytosis (Supplementary Table S5 and Supplementary Fig. S7). We found that proteins associated with caveolin-mediated endocytosis (~10 ± 8, n = 9), clathrin coated vesicles (~17 ± 6, n = 176) and macropinocytosis (~22 ± 7, n = 19) have shorter TMDs than typical plasma membrane proteins (comparing above results in parentheses with those in Fig. 2F). Further, two-tailed heteroscedastic t-tests for above TMD-length distributions yielded p = 0.021 for clathrin coated vesicles vs. caveolin-mediated endocytosis, p = 0.001 for caveolin-mediated endocytosis vs. macropinocytosis, and p = 0.011 for clathrin coated vs. macropinocytosis. These statistically relevant differences (p ≤ 0.05) clearly show that TMD lengths serve as signatures for intracellular sorting mechanisms. Therefore now we were in a position to directly investigate whether viral proteomes consist of TMD length signatures for specific associations with host cell organelles. To be able to do so, first we collected a list of animal viruses for which the primary intracellular pathways (all of which are shown in Fig. 4) involved in their entry into their respective host cells are known^28^,^29^,^30^,^31^,^32^,^33^,^34^,^35^,^36^,^37^,^38^,^39^,^40^,^41^,^42^,^43^,^44^,^45^,^46^. Table 3 shows the collected list of animal viruses, along with the names of specific proteins in their proteomes that are experimentally established to play a key role in their entry.

### Functional application of TMD lengths serving as signatures of organismal complexity towards animal viral transport mechanisms in host cells

**Figure 5:**
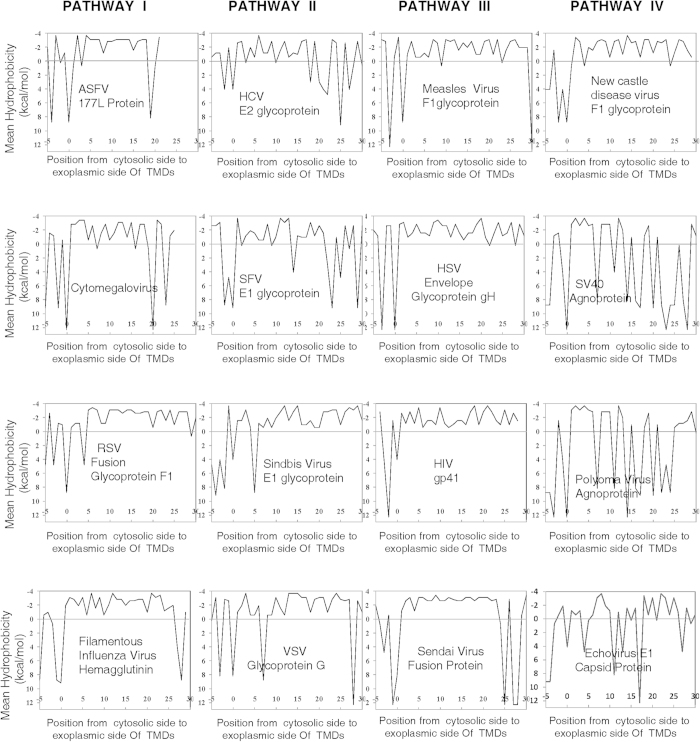
Hydrophobocity graphs of TMDs of animal viral proteins associated with viral entry through various pathways into host cells. Name of the virus, and member of the viral proteome experimentally identified (from literature) as the key to entry into host cells is given in each plot. **Pathway I:** Mode of entry is established to be through macropinocytosis for African Swine Fever Virus, Filamentous Influenza Virus, Respiratory Syncytial Virus and Cytomegalovirus. **Pathway II:** Mode of entry is established to be through clathrin-mediated endocytosis for Hepatitis C Virus, Sindbis Virus, Semliki Forest Virus and Vesicular Stomatitis Virus. **Pathway III:** Mode of entry is established to be through plasma membrane for Measles Virus, Herpes Simplex Virus, Human Immunodeficiency Virus and Sendai Virus. **Pathway IV:** Mode of entry is established to be through caveolin-mediated endocytosis for New Castle Disease Virus, SV40, Polyoma virus and Echovirus.

The next step was to utilize the methodology developed in this work to calculate TMD lengths of each of the specific proteins for each of the animal viruses listed in Table 3. In order to do this, we first obtained hydrophobicity plots for TMD lengths for each of these specific proteins as shown in Fig. 5 (a couple of plots are also shown in supplementary Figs S8A and S8B – they were not included in Fig. 5 to maintain visual symmetry in the main figure). For convenience of interpretation, Fig. 5 also shows the experimentally known entry pathway corresponding to each virus within each hydrophobicity plot. TMD lengths predicted from these hydrophobicity plots are listed in Table 3 in the fifth column, with the last (sixth) column showing mean ± standard deviation of viral proteins’ TMD lengths. Close observation of these results yields a surprisingly solid result supporting the idea that TMD lengths of animal viral proteins do indeed serve as signatures of viral entry mechanisms into their respective host cells. With the sole exception of clathrin-mediated – and caveolin-mediated – endocytosis, there is a clear statistical difference in TMD lengths of viral proteins utilizing different cellular entry pathways (see also supplementary Fig. S8C).

### Functional application of TMD lengths serving as signatures of organismal complexity towards viral replication in plants

**Figure 6:**
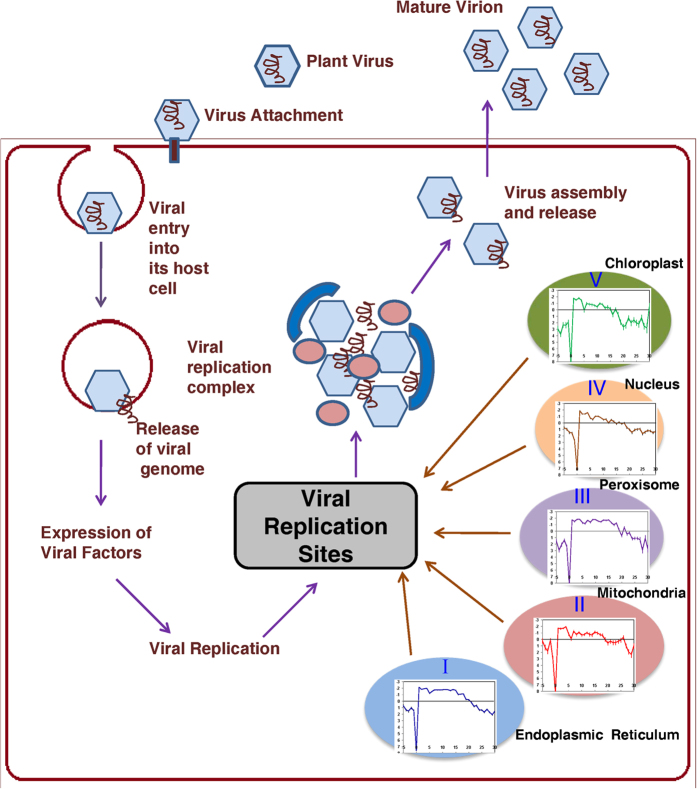
Different virus replication sites for plant viruses. In plants several organelles serve as replication sites of virues. The schematic broadly categorizes these replication sites into ER, mitochondria, peroxisomes, chloroplast and nucleus.

Encouraged by the results obtained above, we decided to explore whether TMD lengths can serve as signatures of viral transport mechanisms for plant viruses also. To our surprise, we found that the literature on plant viral transport mechanisms is quite sparse – however, we found several reports on plant viral replication sites inside plant cells^47^,^48^,^49^,^50^,^51^,^52^,^53^,^54^,^55^. Therefore, based on our literature survey, we compiled a broad classification of replication sites important for viral replication in plant cells, as shown in Fig. 6.

**Figure 7:**
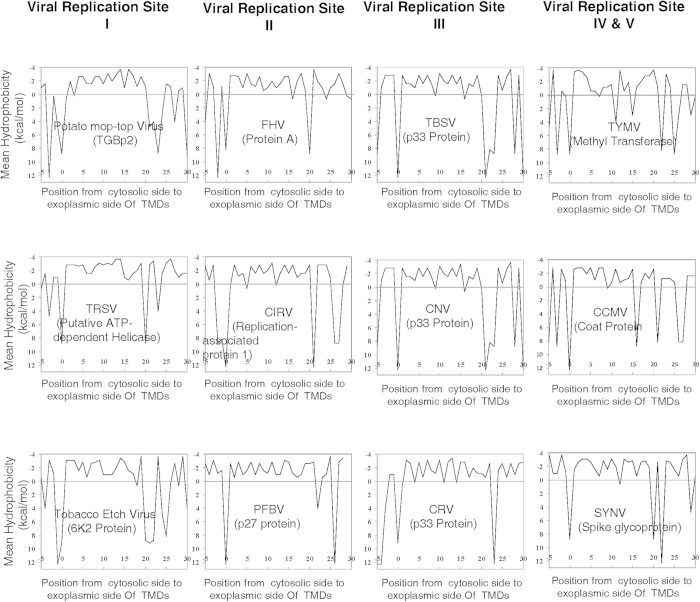
Hydrophobocity graphs of TMDs of plant viral proteins associated with viral replication in plant cells. Name of the virus, and member of the viral proteome experimentally identified as the key to replication in the host cells is given in each plot. Viral Replication Site I: ER serves as the replication site for Potato-mop-top Virus, Tomato Ringspot Virus and Tobacco Etch Virus in plant cells. Viral Replication Site II: Mitochondria serve as the replication sites for Flock House Virus, Carnation Italian Ringspot Virus and Pelargonium Flower Break Virus in plant cells. Note that while Flock House is not a plant virus, it has been shown to replicate at the mitochondria of plant cells. Viral Replication Site III: Peroxisomes serve as replication sites for Tomato Bushy-Stunt Virus, Cucumber Necrosis Virus and Cymbidium Ringspot Virus in plant cells. Viral Replication Site IV: Chloroplasts serve as replication sites for Turnip Yellow Mosaic virus, Cowpea Chlorotic Mottle Virus. It is important to note that the nucleus serves as the replication site (i.e. site V) for Sonchus Yellow Net Virus – this hydrophobicity plot is not shown to maintain visual symmetry in the figure.

Next, as done earlier, we collected a list of plant viruses (including Flockhouse virus, which is not a plant virus, but is known to be able to replicate in plant cells^55^) for which the primary replication sites (shown in Fig. 6) in their respective host cells are known^47^,^48^,^49^,^50^,^51^,^52^,^53^,^54^,^55^. Table 4 shows the collected list of plant viruses, along with the names of specific proteins in their proteomes that are experimentally established to play a key role in viral replication. The next step was to utilize the methodology developed in this work to calculate TMD lengths of each of the specific proteins for each of the plant viruses listed in Table 4. In order to do this, we first obtained hydrophobicity plots for predicting TMD lengths of each of these specific proteins as shown in Fig. 7 (a couple of plots are also shown in supplementary Figs S8D and S8E – they were not included in Fig. 7 to maintain visual symmetry in the main figure). For convenience of interpretation, Fig. 7 also shows the experimentally known replication site corresponding to each virus within each hydrophobicity plot. TMD lengths predicted from these hydrophobicity plots are listed in Table 4 in the fifth column, with the last (sixth) column showing mean ± standard deviation of viral proteins’ TMD lengths. Clearly, TMD lengths of viral replication proteins are not able to distinguish between replication sites of plant viruses with the exception of those involved in replication at the chloroplast (see also supplementary Fig. 8F). However, in the case of chloroplasts also, data is not populated enough (“n” is only 2) to be able to make a strong conclusion. Nevertheless, this analyses of TMD lengths towards gaining insights into plant viral infection mechanisms is, to our knowledge first of its kind, and could be quite promising in future especially when applied to larger data sets on viral replication and transport mechanisms in plants.

## Discussion

The objective of this study was to determine the importance of length of transmembrane domains (TMDs) of single span (bitopic) membrane proteins from an evolutionary perspective. Multi-span membrane proteins were not included since it is not straight forward to identify specific (groups of) residues interacting with membrane lipids in addition to the fact that polytopic membrane proteins usually have active sites mostly buried within the transmembrane helical bundles. Investigations in the last few decades indicate that most of the important protein-protein interactions or protein-lipid interactions take place due to the involvement of transmembrane helices of bitopic membrane proteins instead of polytopic membrane proteins^12^,^13^. The purpose and importance of our work was to proceed beyond pure sequence analysis of single span membrane proteins and to consider implications of TMD lengths for the organellar systems. Here it is important to note that our work evolved very serendipitously while trying to reproduce and extend the results of an elegant study published by Sharpe *et al.*^11^ – in fact it was essential to reproduce their earlier results with our extended data sets (for fungi and vertebrates) to serve as strong positive (computational) controls. Additionally, a close comparison of our results on TMD lengths in only plants showed remarkable agreement with those reported in a recent study^56^. In spite of analyzing independent datasets, our results on plant TMDs (Figs 2B and 3B) confirm the findings of Nikolovski *et al.*^56^ on TMD lengths of proteins associated with ER, Golgi/TGN and plasma membranes in plant cells (their Fig. 4A,B). Our study further extends observations on statistically significant differences in organellar TMD lengths with the inclusion of data on proteins associated with nuclear membranes in plant cells. While reproducibility of earlier findings with independent and expanded datasets is indeed promising by itself, to our knowledge our work here is the first of its kind comprehensive and simultaneous analyses of the available eukaryotic proteomes with different eukaryotic organisms (fungi, plants, non-mammalian vertebrates and mammals – the ordering was chosen by us in view of increased organismal complexity). We find that TMD lengths serve as signatures of the specific organelles in which the corresponding transmembrane proteins reside in different eukaryotic cells. Plasma membrane TMD lengths of all the organisms are longer than those from the TMDs of intracellular organelle membranes, and interestingly, we found that TMD lengths of plasma membrane proteins were similar for all organisms – indicating similar thickness of bilyaers in plasma membranes and supporting a common origin of these eukaryotic cell boundaries^57^. At the same time, there is no relationship in sequence and function of the plasma membrane proteins of all these organisms. The significance of our results is well supported by results indicating that localization of membrane proteins neither depend on their sequence homology nor structural features—the only feature reported to influence localization is length of their TMDs^5^. Interestingly, these results on membrane proteins strongly support recently emerging views on “secularity” of amino acid residues in soluble proteins – composition of primary sequences, in terms of percentage occurrence of amino acids relative to each other (reflected here by TMD lengths identified by hydropathy plots based on relative occurrence of “stretches” of hydrophobic and hydrophilic), rather than the actual sequence in which the residues appear, play a key role in obtaining functional folded proteins^58^,^59^,^60^,^61^,^62^. We have also explored the scope of our results from applicational perspectives by applying our methodology for specifically investigating intracellular and viral transport mechanisms. To our pleasant satisfaction, we find that TMD lengths serve as signatures not only for intracellular transport mechanism native to eukaryotic cells, but also provide clear indications of possible viral transport mechanisms, especially for animal viruses. Future applications of methodologies developed in this work may provide great assistance in designing well-directed experiments for investigating intracellular transport mechanisms utilized by viruses, whose mechanisms are not yet known, but whose proteomes can be determined by using modern experimental proteomic tools. Further, our results on TMD lengths of bitopic proteins, approximated as helices and representing bilayer thickness, is also a strong addition to the growing literature on geometrical interpretations of molecular interactions especially pertaining to membranes and proteins in biology^63^,^64^,^65^.

### Conclusions: A new perspective on organismal complexity

Finally, we wish to emphasize that our results provide a unique and novel evolutionary perspective. Two contrasting, but highly appealing, evolutionary inferences can be made from our results. From Fig. 3E it can be inferred that the next major step in evolution of complexity in eukaryotic cells is a further decrease in differences between TMD lengths, and hence bilayer thickness, of plasma membranes and intracellular organellar systems. From a philosophical standpoint, it appears analogous to homogenization of differences between various compartments of the cell and cell boundary – reflective of evolution of the society in general. Alternatively, Fig. 3E may indicate that an evolutionary saturation in differences between bilayer thickness of plasma membranes and intracellular organellar systems has been reached or will be reached soon. Beyond this, a new evolutionary cycle may begin with simpler eukaryotic cells originating again and leading to further complex systems. Regardless of which of the above two are correct, they are equally thought provoking and open up a fresh avenue towards views on cellular complexity and evolution.

## Methodology

### Data Collection

In our study, we have done comparative analysis of transmembrane domains of membrane proteins (n = 68,281) of different organelles from discrete subcellular locations. We collected datasets of bitopic transmembrane proteins from the best studied eukaryotic genomes—therefore reference proteins for fungi were collected from *Saccharomyces cerevisiae* for the computational analysis of TMD proteins of their different organelles (Endoplasmic Reticulum, Golgi, TGN/Endo and Plasma membrane). Reference proteins for plants were collected from *Arabidopsis thaliana* for the computational analysis of TMD proteins of ER, Golgi, Nucleus, Mitochondria, Peroxisomes, Chloroplast and Plasma Membrane. Reference proteins for non-mammalian vertebrates and mammals were collected from *Gallus gallus* and *Homo sapiens* respectively for TMD analysis of all their organelles. Reference proteins are those proteins whose TMD organelle localisation and TMD span (start and end) definitions are well known – this original dataset of all reference proteins comprised of 394 sequences. Accession numbers of reference proteins for fungi and non-mammalian vertebrates were collected from literature^11^ whose organelle residences and topology were known and best studied. Accession numbers of reference proteins for plants were collected from Plant Proteome Database, ARAMEMNON Database and AT_CHLORO Database. For mammals accession numbers were collected from literature searches and LOCATE DATABASE. Accession numbers for membrane proteins involved in clathrin-mediated endocytosis, macropinocytosis and caveolin-mediated endocytosis were collected from National Centre for Biotechnology Information. Accession numbers of membrane proteins of animal and plant viruses (enveloped and non-enveloped) were collected from Viral Zone Database and National Centre for Biotechnology Information. All accession numbers and related information is provided in supplementary information (with tables).

### Collection of orthologous membrane proteins and transmembrane protein orientation

Size of our initial dataset was somewhat limited due to selection of only those membrane proteins whose organelle location and topology were known in literature (n = 394). Thus, in order to increase the dataset (i.e. number of sequences) for our TMD analysis we did BLAST to collect orthologous proteins for each organism. We collected orthologous proteins from 162 fungal-, 32 plant-, 17 non-mammalian vertebrate- and 90 mammalian- genomes by using BLAST to augment the sequence information (n = 68,281). The cut-off stringency for BLAST was smaller than E = 10^−10^. After collecting all the sequences (n = 68,281), TMHMM server used to predict the transmembrane protein orientation in the reference protein and the orthologous proteins. The hydrophobic spans were aligned from the cytosolic side to the exoplasmic side.

### Screening of TMD span in membrane protein sequences

We used Goldman-Engelman-Steitz (GES) scale because it is based on thermodynamic measurement, rather than a statistical one. Therefore for preparing hydrophobicity graphs we used GES hydrophobicity scale. To avoid biasness and to ensure that our results are not hydrophobicity-scale-dependent, we also used the Kyte-Doolittle scale for our analyses. Then we analysed the “rough” position of the TMDs in all the membrane protein sequences by giving window size of 18 residues initially for all the organelles (Golgi, Endoplasmic Reticulum, TGN/Endosomes, Nucleus, Chloroplast, Peroxisomes, Mitochondria and Plasma Membrane). We defined the initial 18 residue window to be the one that is the most hydrophobic in the transmembrane region, irrespective of how many hydrophilic residues were present within it (and their relative location). This step also ensured an additional check of the presence of a TM span in the orthologous sequences. The TMD spans identified in this screening step were then catalogued along with their flanking residues (i.e. those residues that are next to both cytosolic and exoplasmic edges of the “rough” TMDs). A maximum of 8 residues on both sides were considered as flanking residues. Thus, sequences emerging out of this step of analysis had a maximum possible length of 34 residues (8 + 18 + 8) and a minimum possible length of 26 residues (8 + 18 or 18 + 8).

### Reduction in sequence redundancy based on sequence similarity

In order to ensure that our analyses were not biased because of presence of closely related sequences in our orthologous collection, we used BLASTClust to screen for sequence redundancy in our dataset (i.e. sequences obtained from the last step of the previous screening). Using BLASTClust we checked the similarity between reference and their corresponding orthologous protein sequences, and also among different orthologous proteins of the same reference protein. The aim was to collect only those protein sequences from each group which were not more than 30% similar to each other. This clustering process was performed on the TMD region along with their flanking sequences. Final numbers of non-redundant sequences are shown in Table 1.

### Refinement of TMD span – Defining “Start” and “End” edges of TMDs

The next, but the most crucial step, was refinement of TMD span edges (start and end points). In the previous “rough” screening for TMDs, we had allowed hydrophilic residues if they are followed by sufficiently hydrophobic residues, and if they were not followed by hydrophobic residues then they were chopped off the edge of TMD span. It is challenging to deal with the edge cases of TMDs, especially in case of individual hydrophilic residues appearing in the middle of the core region of TMDs (thereby having negligible effects on overall hydrophobicity scores). However, hydrophilic residues at the edge of TMD spans help to define the edge precisely and are not included in TMDs. If we follow the above rule then the end points of the TMD span, i.e. edges, would contract (i.e. the length of the TMD span would reduce) if the hydrophilic residues on the edges are not surrounded by sufficiently hydrophobic residues (e.g. three hydrophobic residues after and three hydrophobic residues before any hydrophilic residue). Further, if hydrophilic residues are surrounded by sufficiently hydrophobic residues, the edges of 18 residue region would expand resulting in the difference in TMD lengths among different sequences. Essentially, the most important step is to find out the hydrophilic to hydrophobic transition (TM/aqueous vs buried) in the sequences. On the basis of above, all the protein sequences from each set (e.g. for each organism and each organelle) were aligned at the position where a sharp change in hydopathy occurred – the cytosolic end of hydrophobic region was assumed as position one (01) while doing this. The next challenge was to consistently define the end of transmembrane spans in sequences. Here it is important to note that while TMHMM is excellent at identifying TM spans, however, the exact end points can vary – even if the variation is only by a couple of residues only, it is not precise. To overcome this limitation, we recognized that once all the ends are refined consistently (i.e. applying exactly the same series of steps) based on hydrophobicity and the presence of charges, all of the features became much sharper (at both ends of the span). Therefore instead of using TMHMM server for defining TM span we wrote a refinement algorithm (executed in MATLAB, Mathworks Inc.). Figure S9 shows the algorithm developed and used by us as a flow chart (with description). Implementation of this algorithm enabled us to align protein sequences in a given dataset (e.g. an organelle set) at the positions where a sharp change in hydropathy occurred. Thus, after implementation of this algorithm to our sequences, we were able to plot hydrophobicity graphs of each of the TMD sequences from different datasets, belonging to fungi, plants, non-mammalian vertebrates and mammals and their different subcellular locations/organelles (Plasma Membranes, ER, Golgi, TGN/Endosomes, Mitochondria, Chloroplast, Nucleus, Peroxisomes). Additionally, the above approach was applied to obtain hydrophobicity graphs of protein sequences (a) involved in different endocytic pathways, and, (b) from animal and plant viruses.

### Statistical analyses for comparing TMD lengths and distributions

We performed **t-tests** to test the significance of differences (or lack thereof) in TMD lengths among different organelles and report the p-values obtained. To confirm our findings rigorously, we also performed **Kullback-Leibler divergence tests** with distributions of the TMD lengths. KL Divergence measure (KLDM) was calculated for all combinations of organelle sets in each organism. Since the measure is asymmetric, it gives different values when a distribution X is compared to Y with X as the base distribution vs with Y as the base distribution. The symmetric version of KLDM would simply be the average of KLDM for X vs Y and Y vs X measures. Briefly, KL divergence method function takes three arguments

1. X: the set of values.
2. P1: First probability distribution.
3. P2: Second probability distribution.

Since KLDM calculation involves logarithm of probabilities (relative frequencies), any entries that have no occurrences in the two distributions being compared cause the calculation to fail. Since our intent was pair-wise comparisons of all types within each organelle, we had to trim the data so that all probability entries considered for KLD measure were non zero.

## Additional Information

**How to cite this article**: Singh, S. and Mittal, A. Transmembrane Domain Lengths Serve as Signatures of Organismal Complexity and Viral Transport Mechanisms. *Sci. Rep.* **6**, 22352; doi: 10.1038/srep22352 (2016).

## Supplementary Material

Supplementary Information

## Figures and Tables

**Table 1:** Mean hydrophobic lengths of TMDs of different organelles.

Organism	ER	Golgi	TGN/endo/Nucleus	PM
Fungi	22.60 ± 6.4 n = 2446	20.25 ± 5.8 n = 4686	23.28 ± 5.7 n = 865	24.80 ± 8.3 n = 785
Plants	22.80 ± 6.7 n = 1813	23.80 ± 7.7 n = 3197	19.40 ± 7.8 n = 801	24.47 ± 5.3 n = 2519
Non-Mammalian Vertebrates	23.13 ± 7.1 n = 327	19.86 ± 5.9 n = 823	24.26 ± 6.4 n = 429	24.00 ± 6.8 n = 1072
Mammals	23.65 ± 6.1 n = 1082	21.44 ± 6.9 n = 1657	25.90 ± 7.4 n = 644	24.95 ± 6.4 n = 3113

**Table 2:** Statistical analyses of differences in TMD lengths between different organelles using t-tests and Kullback-Leibler Divergence Measure (KLDM) – Fungi, Plants, Non-Mammalian Vertebrates and Mammals.

Fungi	ER	Golgi	TGN/endo	PM
ER	—	p = 6.3 × 10^−51^ KLDM = 0.5155	p = 0.004 KLDM = 0.0787	p = 4 × 10^−12^ KLDM = 0.157
Golgi	p = 6.3 × 10^−51^ KLDM = 0.4208	—	p = 1 × 10^−42^ KLDM = 0.4599	p = 2.9 × 10^−22^ KLDM = 0.4953
TGN/endo	p = 0.004 KLDM = 0.0779	p = 1 × 10^−42^ KLDM = 0.4604	—	p = 8.2 × 10^−6^ KLDM = 0.166
PM	p = 4 × 10^−12^ KLDM = 0.1636	p = 2.9 × 10^−22^ KLDM = 0.8733	p = 8.2 × 10^−6^ KLDM = 0.1927	—
**Plants**	**ER**	**Golgi**	**Nucleus**	**PM**
ER	—	p = 8.1 × 10^−7^ KLDM = 0.0684	p = 1.6 × 10^−25^ KLDM = 0.3945	p = 6.9 × 10^−18^ KLDM = 0.2841
Golgi	p = 8.1 × 10^−7^ KLDM = 0.0818	—	p = 1.9 × 10^−43^ KLDM = 0.4499	p = 4 × 10^−4^ KLDM = 0.4539
Nucleus	p = 1.6 × 10^−25^ KLDM = 0.4884	p = 1.9 × 10^−43^ KLDM = 0.502	—	p = 8.5 × 10^−58^ KLDM = 0.942
PM	p = 6.9 × 10^−18^ KLDM = 0.2578	p = 4 × 10^−4^ KLDM = 0.3519	p = 8.5 × 10^−58^ KLDM = 0.7979	—
**Non-Mammalian Vertebrates**	**ER**	**Golgi**	**TGN/endo**	**PM**
ER	—	p = 7.4 × 10^−13^ KLDM = 0.4353	p = 0.024 KLDM = 0.4005	p = 0.051 KLDM = 0.2239
Golgi	p = 7.4 × 10^−13^ KLDM = 0.4018	—	p = 6.9 × 10^−30^ KLDM = 0.9439	p = 5.7 × 10^−43^ KLDM = 0.6351
TGN/endo	p = 0.024 KLDM = 0.4154	p = 6.9 × 10^−30^ KLDM = 0.8613	—	p = 0.485 KLDM = 0.1081
PM	p = 0.051 KLDM = 0.2528	p = 5.7 × 10^−43^ KLDM = 0.5843	p = 0.485 KLDM = 0.1181	—
**Mammals**	**ER**	**Golgi**	**TGN/endo**	**PM**
ER	—	p = 4.4 × 10^−18^ KLDM = 0.4732	p = 1.3 × 10^−10^ KLDM = 0.4936	p = 2.9 × 10^−9^ KLDM = 0.205
Golgi	p = 4.4 × 10^−18^ KLDM = 0.439	—	p = 7.1 × 10^−37^ KLDM = 0.434	p = 8.8 × 10^−63^ KLDM = 0.417
TGN/endo	p = 1.3 × 10^−10^ KLDM = 0.5438	p = 7.1 × 10^−37^ KLDM = 0.4457	—	p = 0.003 KLDM = 0.174
PM	p = 2.9 × 10^−9^ KLDM = 0.2016	p = 8.8 × 10^−63^ KLDM = 0.3761	p = 0.003 KLDM = 0.1678	—

**Table 3:** Viral entry pathways of animal viruses and TMD lengths (predicted from hydrophobicity plots) of proteins known to play a key role in viral entry.

S. No.	Enveloped Virus (Protein)	Known* Entry Mode	Reference(s) for*	Predicted TMD Length(s)	Mean ± Std
1	HIV (gp41)	PM	33,45	30	28.25 ± 2.36
2	Herpes Simplex (Env glycoprotein gH)		33,45	28	
3	Measles (Hemagglutinin)		33,45	30	
4	Sendai (Fusion Protein)		33,45	25	
5	Hepatitis C (E2 glycoprotein)	CME	29,30	17	10.80 ± 5.12
6	Sindbis (E1 glycoprotein)		28	5	
7	Vesicular Stomatitis (Glycoprotein G)		31,32	7	
8	Semliki Forest (E1 glycoprotein)		33	15	
9	Equine Infectious Anemia (Outer membrane protein)		34	10	
10	Vaccinia (A21 protein)	MPE	33,46	8	20.67 ± 7.06
11	Filamentous Influenza (Hemagglutinin)		38	27	
12	Cytomegalovirus (Env Glycoprotein H)		37	20	
13	Respiratory Syncytial virus A (F1 protein)		36	27	
14	African Swine Fever (177L)		35	19	
15	Ebola (Envelope Glycoprotein)		39, 40, 41	23	
16	SV 40 (Agnoprotein)	CAME	43	10	13.75 ± 7.50
17	Polyoma (Agnoprotein)		43	10	
18	New Castle Disease (F1 protein)		42	25	
19	Echovirus 1 (Capsid protein)		44	10	

PM – Plasma membrane; CME – Clathrin-mediated endocytosis; MPE – Macropinocytosis; CAME – Caveolin-mediated endocytosis.

**Table 4:** Viral replication sites of plant viruses and TMD lengths (predicted from hydrophobicity plots) of proteins known to play a key role in replication.

S. No.	Plant Virus (Protein)	Known* Replication Site	Reference(s) for*	Predicted TMD Length(s)	Mean ± Std
1	Potato mop-top (TGBp2)	Endoplasmic Reticulum	51	20	19.83 ± 0.41
2	Tomato-ringspot (X2)		51	20	
3	Tobacco-etch (6K2 protein)		48	20	
4	Potato Virus X (TGBp3)		49	20	
5	Maize Dwarf Mosaic (6K2)		48	19	
6	Turnip Mosaic (6K2)		51	20	
7	Flock house* (Protein A)	Mitochondria	55	20	20.67 ± 0.58
8	Carnation Italian Ringspot (Replication-associated protein 1)		50	21	
9	Pelargonium Flower Break (p27 protein)		54	21	
10	Tomato Bushy Stunt (p33)	Peroxisome	47	21	21.67 ± 1.15
11	Cymbidium Ringspot (Movement protein)		51	23	
12	Cucumber Necrosis (p33)		52	21	
13	Turnip Yellow Mosaic (Methyl transferase)	Chloroplast	51	11	13.00 ± 2.83
14	Cowpea chlorotic mottle (coat protein)		53	15	
15	Sonchus Yellow Net (M2 protein)	Nucleus	51	20	20

^*^Flock house virus is actually not a plant virus, but can replicate in plant cells.
