# Supplementary material for: Transmembrane Domain Lengths Serve as Signatures of Organismal Complexity and Viral Transport Mechanisms
Source: Sci Rep. 2016 Mar 1;6:22352. doi: 10.1038/srep22352 (PMC4772119; doi:10.1038/srep22352)
Supplement: Supplementary Information [file srep22352-s1.pdf]

Supplementary Information

Transmembrane Domain Lengths Serve as Signatures of Organismal Complexity and Viral Transport Mechanisms

by

Snigdha Singh and Aditya Mittal

Index for Supplementary information

S. No.	Description of Contents	Page Nos.
1	**Figures S1 – S8** with legends	3 – 19
2	Species of fungi	20 – 21
3	**Table S1**	22
4	Species of plants	22
5	**Table S2**	23
6	Species of non-mammalian vertebrates	24
7	**Table S3**	24
8	Species of mammals	25
9	**Table S4**	26 – 27
10	**Table S5**	28 – 36

Supplementary Figure S1

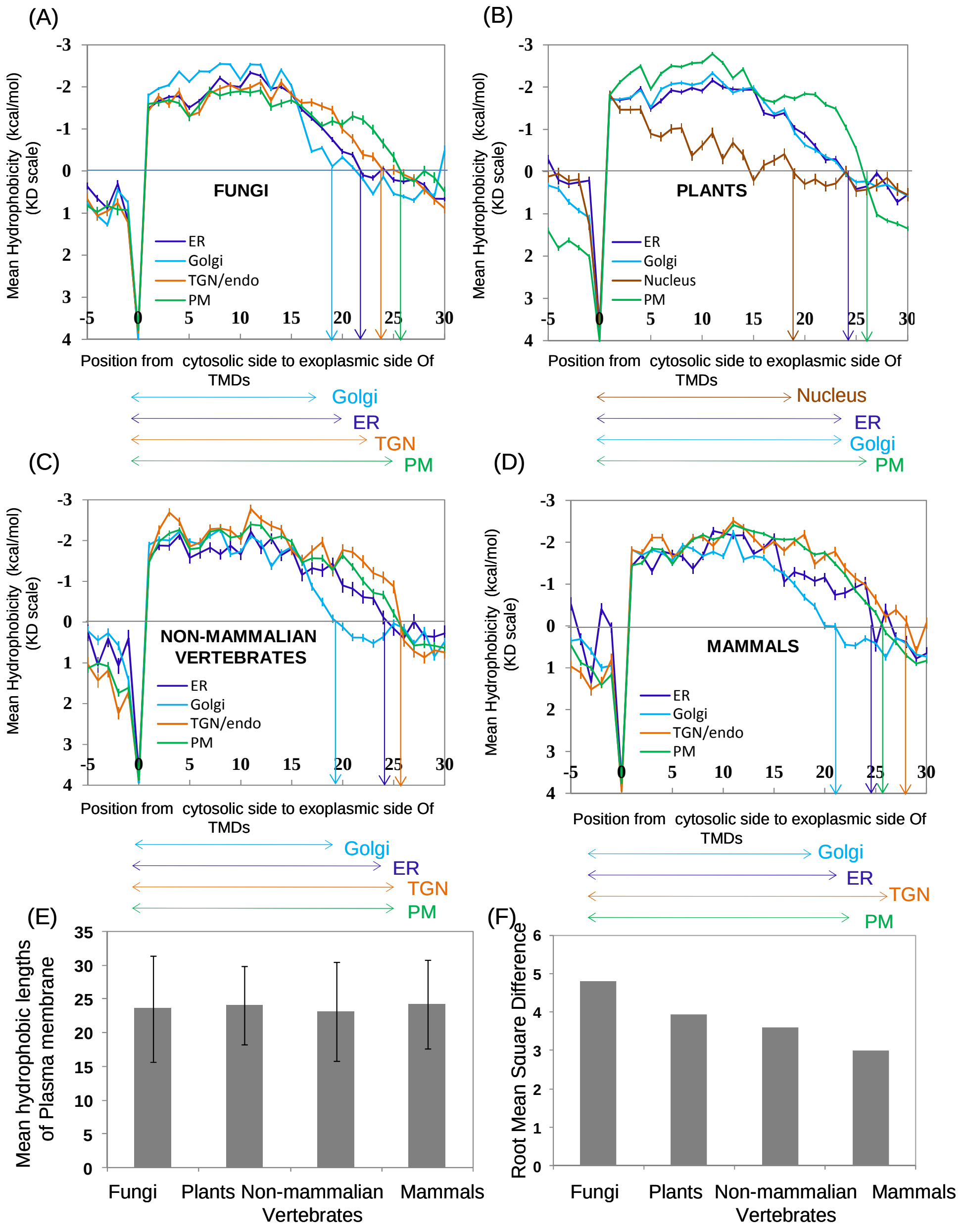

Figure S1: Hydrophobicity profiles of TMD lengths of organelles and plasma membranes in fungi, plants, non-mammalian vertebrates and mammals using the **Kyte-Doolittle scale**. Note that **(A) – (D)** above correspond to main Figs. 2A – 2D respectively. Further, **(E)** above corresponds to main Fig. 2F and **(F)** above corresponds to main Fig. 3E. The analyses shown in main figures was done using the GES scale – this figure confirms that the results found by us are independent of hydrophobicity scales.

Supplementary Figure S2

(A)

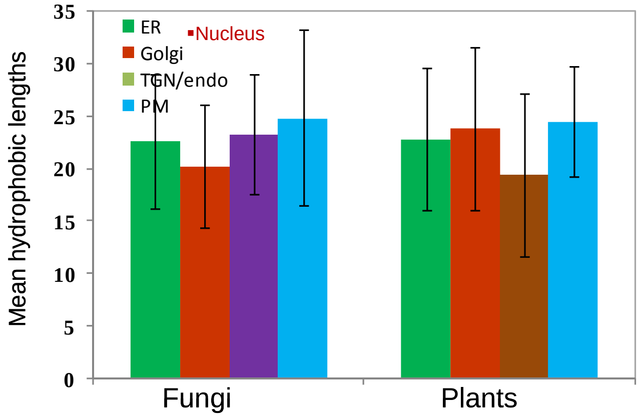

(B)

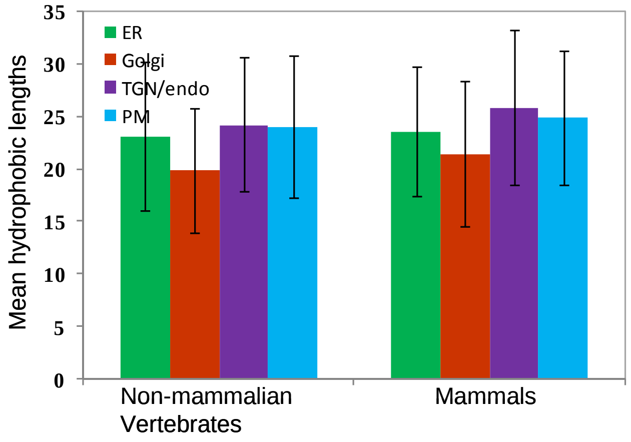

Figure S2:

(A) Mean hydrophobic lengths of ER, Golgi, TGN, Plasma Membranes (PM) in fungi and ER, Golgi, Nucleus, PM in plants.

(B) Mean hydrophobic lengths of ER, Golgi, TGN, PM in non-mammalian vertebrates and ER, Golgi,TGN, PM in mammals.

Supplementary Figure S3 (continued on next page)

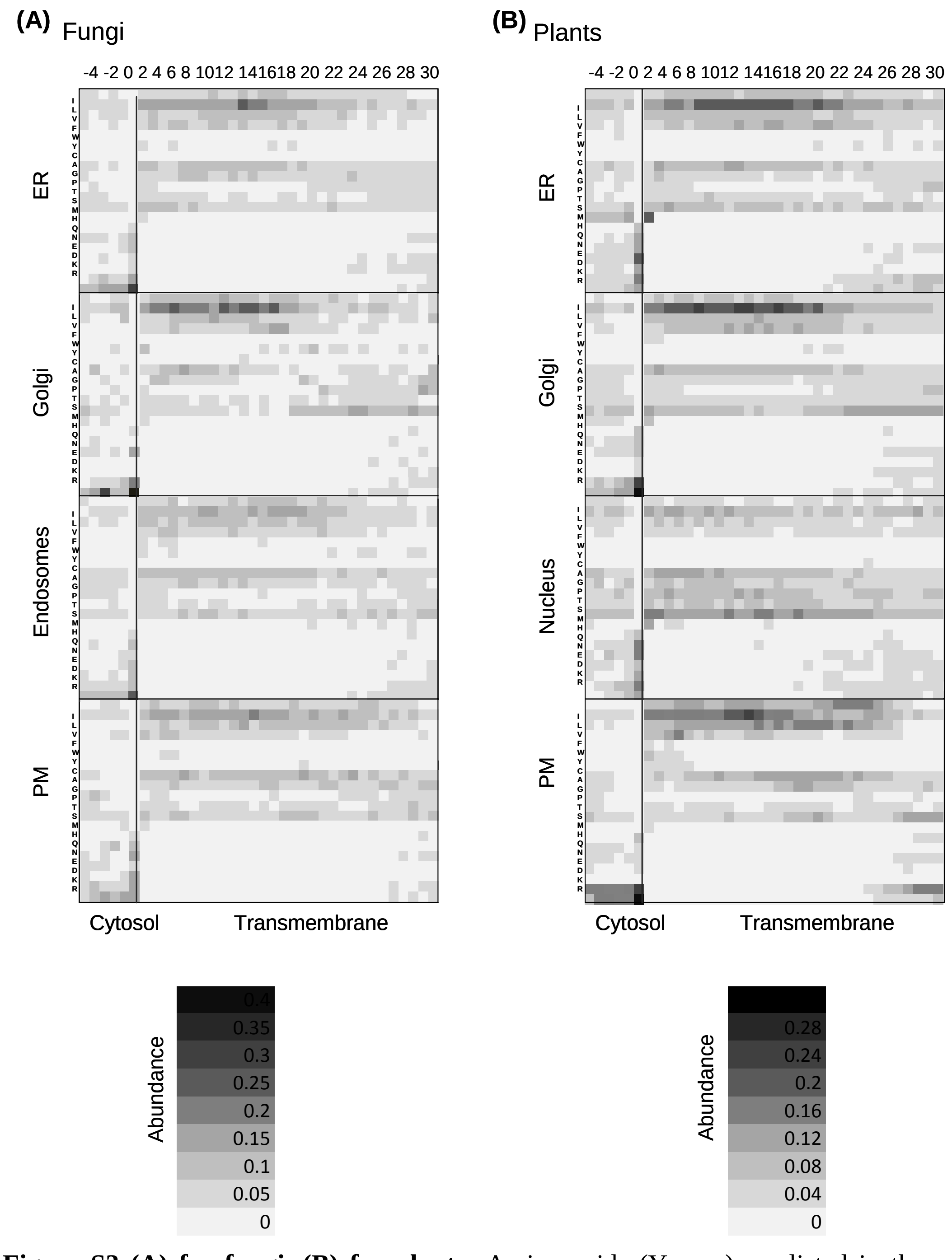

Figure S3 (A) for fungi, (B) for plants: Amino acids (Y-axes) are listed in the order of decreasing hydrophobicity index values. X-axes represent position w.r.t. cytosol. White represents zero and dark grey color a maximum of one. Along the length of the TMDs there is a change in the abundance of hydrophobic residues.

Supplementary Figure S3 (“Explanation” continued on next page)

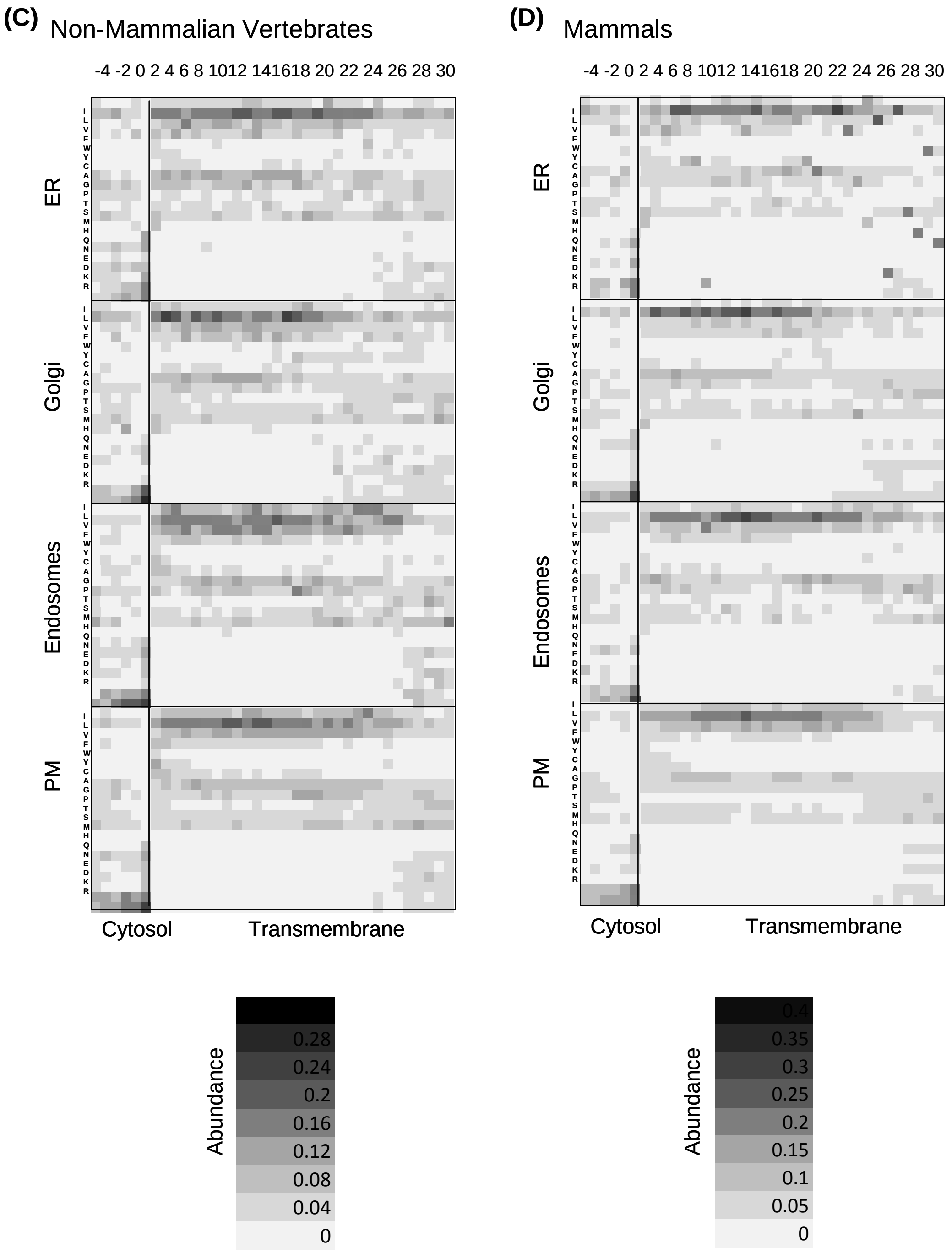

Figure S3 (C) for non-mammalian vertebrates, (D) for mammals: Amino acids (Y-axes) are listed in the order of decreasing hydrophobicity index values. X-axes represent position w.r.t. cytosol. White represents zero and dark grey color a maximum of one. Along the length of the TMDs there is a change in the abundance of hydrophobic residues.

Supplementary Figure S3 - Explanation

Positional analyses of amino acid compositions of TMDs from different organelles show interesting compositional differences between and along the TMDs. This analysis served as an extremely important computational positive control for our work – we were able to confirm the findings of Sharpe et al. (2010) with a larger dataset (on fungi and vertebrates) as well as the methodology developed in this work. Leucine is found to be more abundant in TMD region of ER, Golgi, TGN/endo and PM but less abundant in cytosolic region. Valine is abundant in TMD region in plasma membrane, TGN/endo. The regions abundant in hydrophobic residues are larger for TGN/endo and plasma membrane proteins than ER and Golgi proteins, indicating a difference in TMD length. Polar residues are abundant in cytosolic regions. (A) In fungi regions enriched in hydrophobic residues are shortest for Golgi proteins. Leucine is most abundant in TMD regions of ER and Golgi. (B) In plants regions enriched in hydrophobic residues are similar in ER and golgi TMDs. (C) In non-mammalian vertebrates regions enriched in hydrophobic residues are shortest for Golgi proteins. Leucine is most abundant in ER rather than all other organelles. (D) In mammals also regions enriched in hydrophobic residues are shortest for Golgi proteins. Regions occupied by hydrophobic residues in membrane proteins of ER, endosomes and plasma membrane are almost similar. **Summary of compositional Analysis**: In fungi, regions enriched in hydrophobic residues are shortest for golgi proteins and longest for plasma membrane proteins indicating a difference in TMD length. Further, different hydrophobic residues were not uniformly distributed through the hydrophobic TMD core. For example, leucine is most abundant in TMD regions of ER and Golgi in fungi. In plants, regions enriched in hydrophobic residues are same for ER and golgi proteins indicating much less difference in TMD length of both the organelles, as also observed from the above hydrophobicity graphs. In plants, leucine is most abundant in TMD regions of ER, golgi and plasma membrane. In non-mammalian vertebrates the regions enriched in hydrophobic residues are longest for endosome proteins rather than plasma membrane proteins indicating a difference in TMD length. Valine is most abundant in the TMD regions of ER and endosomes. Leucine is most abundant in the TMD region of ER in non-mammalian vertebrates as compared to other organelles. In mammals the regions enriched in hydrophobic residues are shortest for golgi proteins compared to all other organelles and plasma membranes. Statistical validity of above results was confirmed by results shown in supplementary Fig. S4.

Supplementary Figure S4

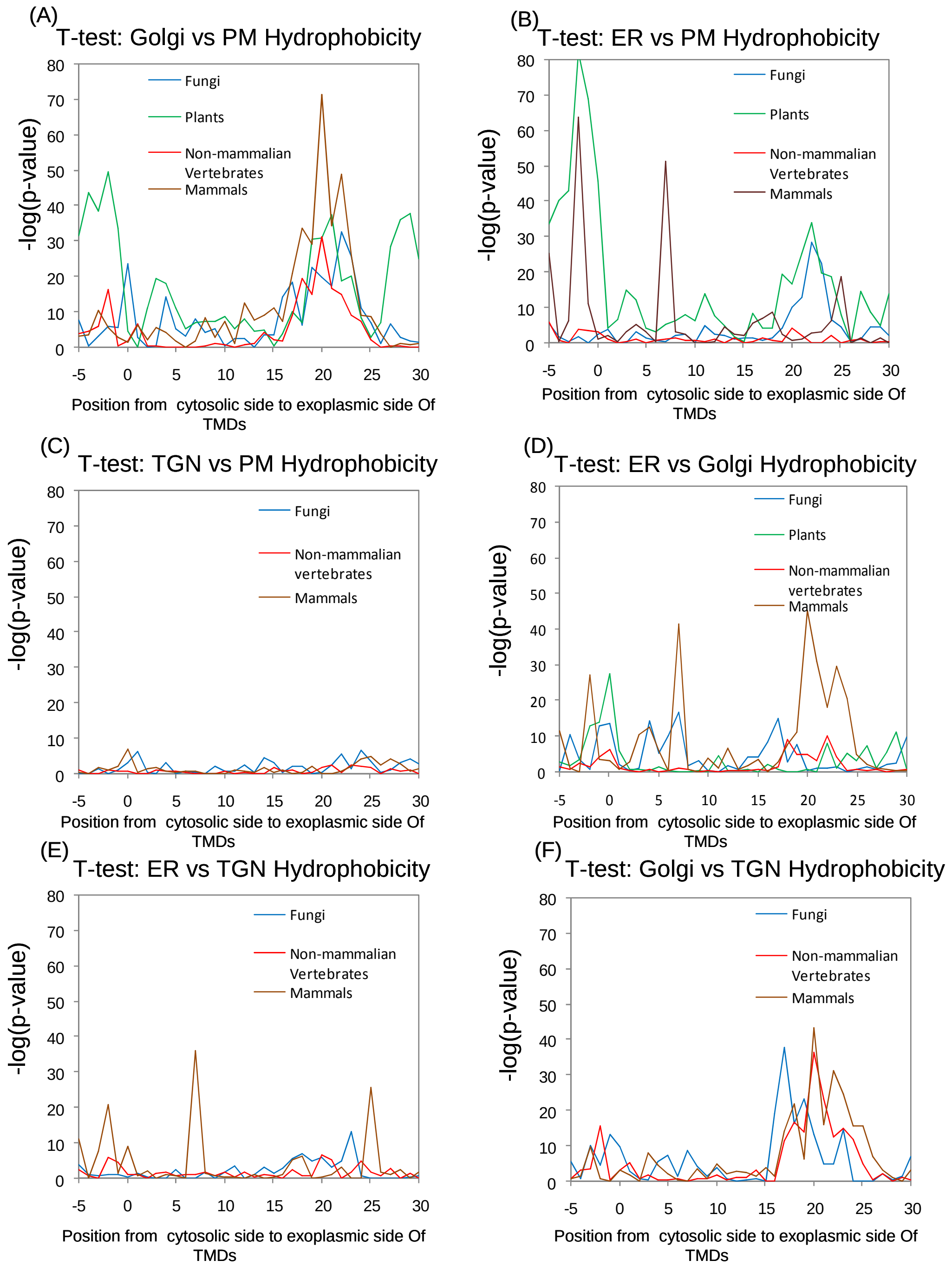

Figure S4: Independent (two-sample) t-tests to compare the mean residue hydrophobicity of residues at positions relative to cytosolic edge of TMDs.

(A) Golgi vs. PM: For fungi the hydrophobicity values of the Golgi and plasma membrane TMDs were highly significantly different between positions 15 and 25. For non-mammalian

vertebrates, the difference between Golgi and plasma membrane TMDs was significant from positions 17 to 25 but this difference was more significant in the case of mammals. In mammals and even in plants there is a significant difference in mean hydrophobicity profiles of amino acid residues relative to their positions i.e. there is a difference in the composition of transmembrane domains between golgi and plasma membrane. **(B) ER vs. PM:** For fungi the hydrophobicity values of ER and plasma membrane TMDs were highly significantly different between positions 18 and 25. For plants and mammals these differences are more significant than fungi and non-mammalian vertebrates. **(C) TGN/endo vs. PM:** In fungi the difference between the mean hydrophobicity profiles of amino acid residues was significant up to some extent but in plants, non-mammalian vertebrates and mammals these differences were not significant. **(D) ER vs. Golgi:** As compared to fungi and non-mammalian vertebrates the difference in mean hydrophobicity profiles of transmembrane domains of Golgi and endoplasmic reticulum in mammals is most significant. **(E) ER vs TGN/endo:** In fungi the difference between the mean hydrophobicity profiles of amino acid residues was significant from positions 15 to 24. **(F) Golgi vs TGN/endo:** In fungi the difference between the mean hydrophobicity profiles of amino acid residues was highly significant from positions 15 to 24. In non-mammalian vertebrates the difference was significant from positions 16 to 26 and in mammal the difference between the hydrophobicity profiles is significant from positions 16 to 29.

Results of comprehensive abundance analysis for each amino acid along the TMDs are shown in Fig. S5.

Supplementary Figure S5 (continued on next page)

(A)

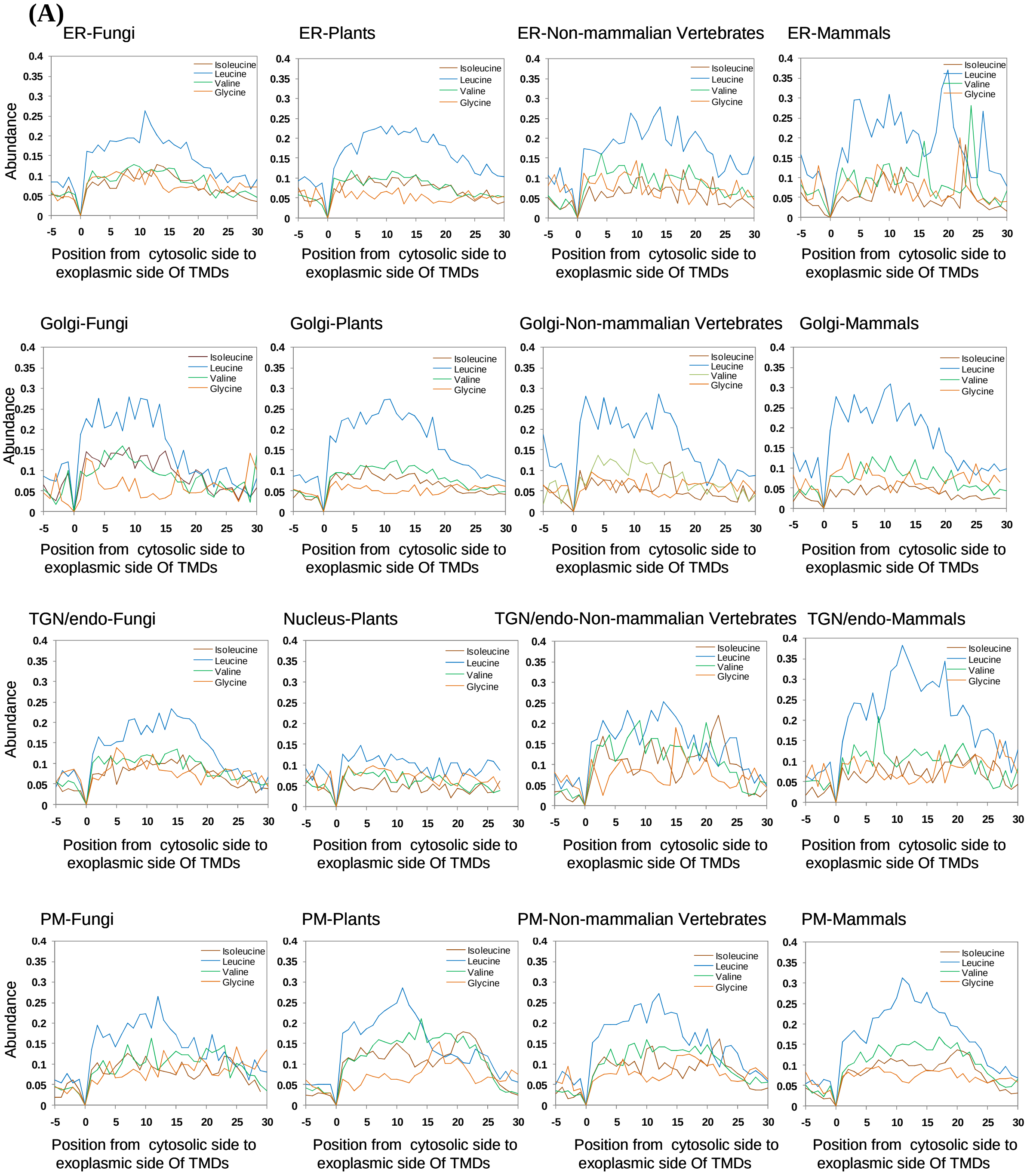

Figure S5: (A) Analysis of the abundance of isoleucine, leucine, valine and glycine along the TMDs from ER, TGN/endo, Golgi, Nucleus and Plasma membrane in fungi, plants, non-mammalian vertebrates and mammals.

Supplementary Figure S5 (continued on next page)

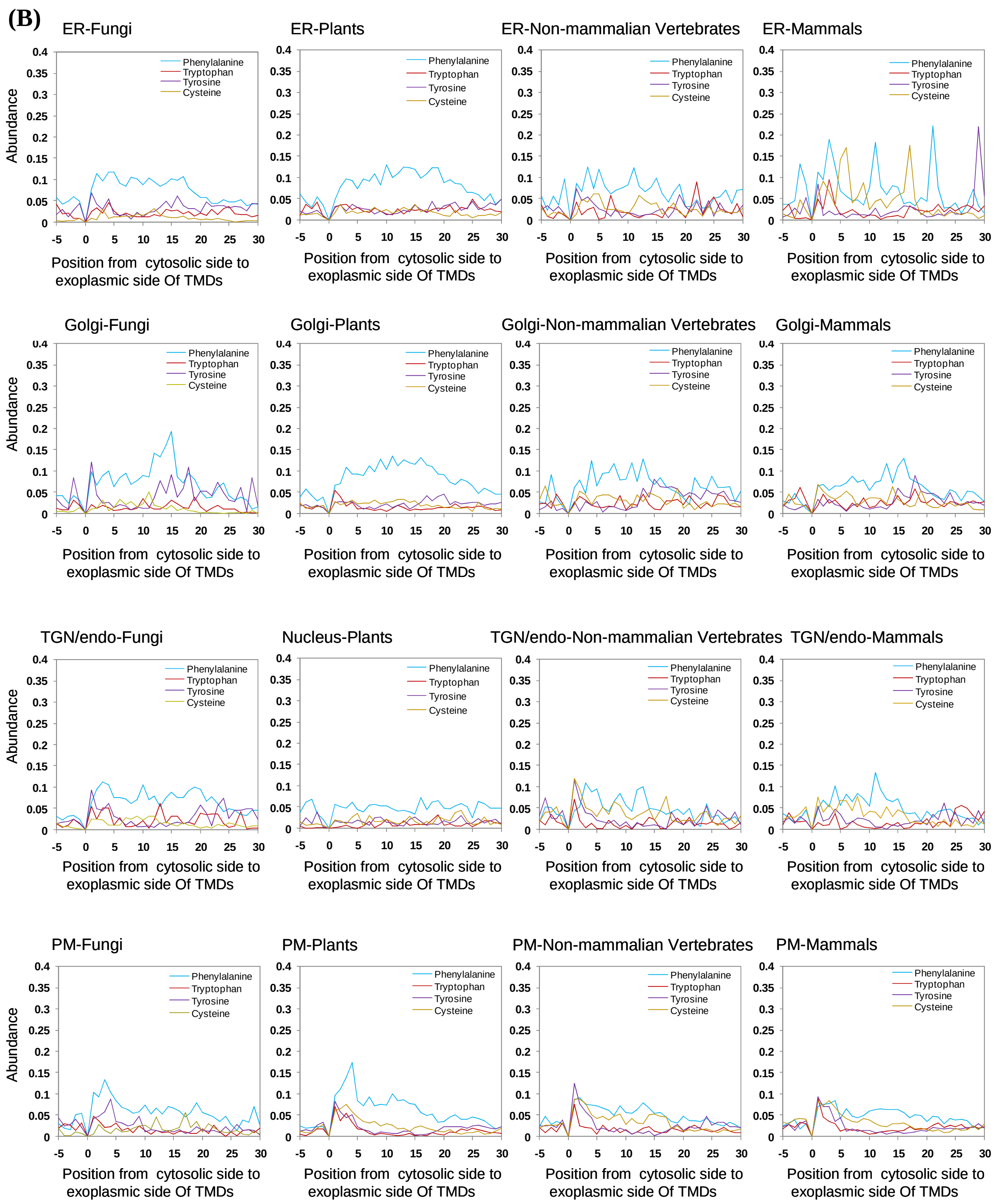

Figure S5: (B) Analysis of the abundance of phenylalanine, tryptophan, tyrosine and cysteine along the TMDs from ER, TGN/endo, Golgi, Nucleus and Plasma membrane in fungi, plants, non-mammalian vertebrates and mammals.

Supplementary Figure S5 (continued on next page)

(C)

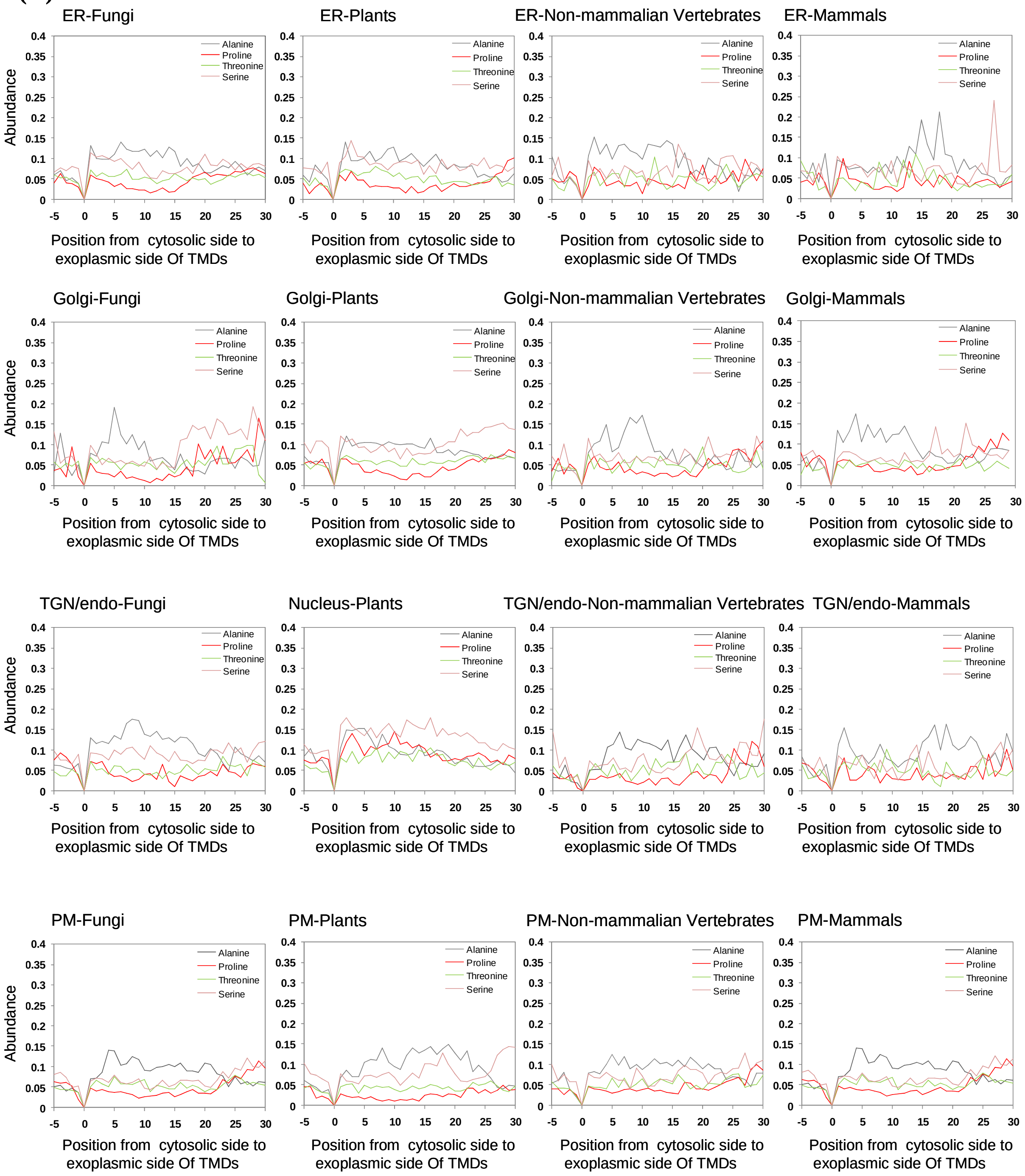

Figure S5: (C) Analysis of the abundance of alanine, proline, threonine and serine along the TMDs from ER, TGN/endo, Golgi, Nucleus and Plasma membrane in fungi, plants, non-mammalian vertebrates and mammals.

Supplementary Figure S5 (continued on next page)

(D)

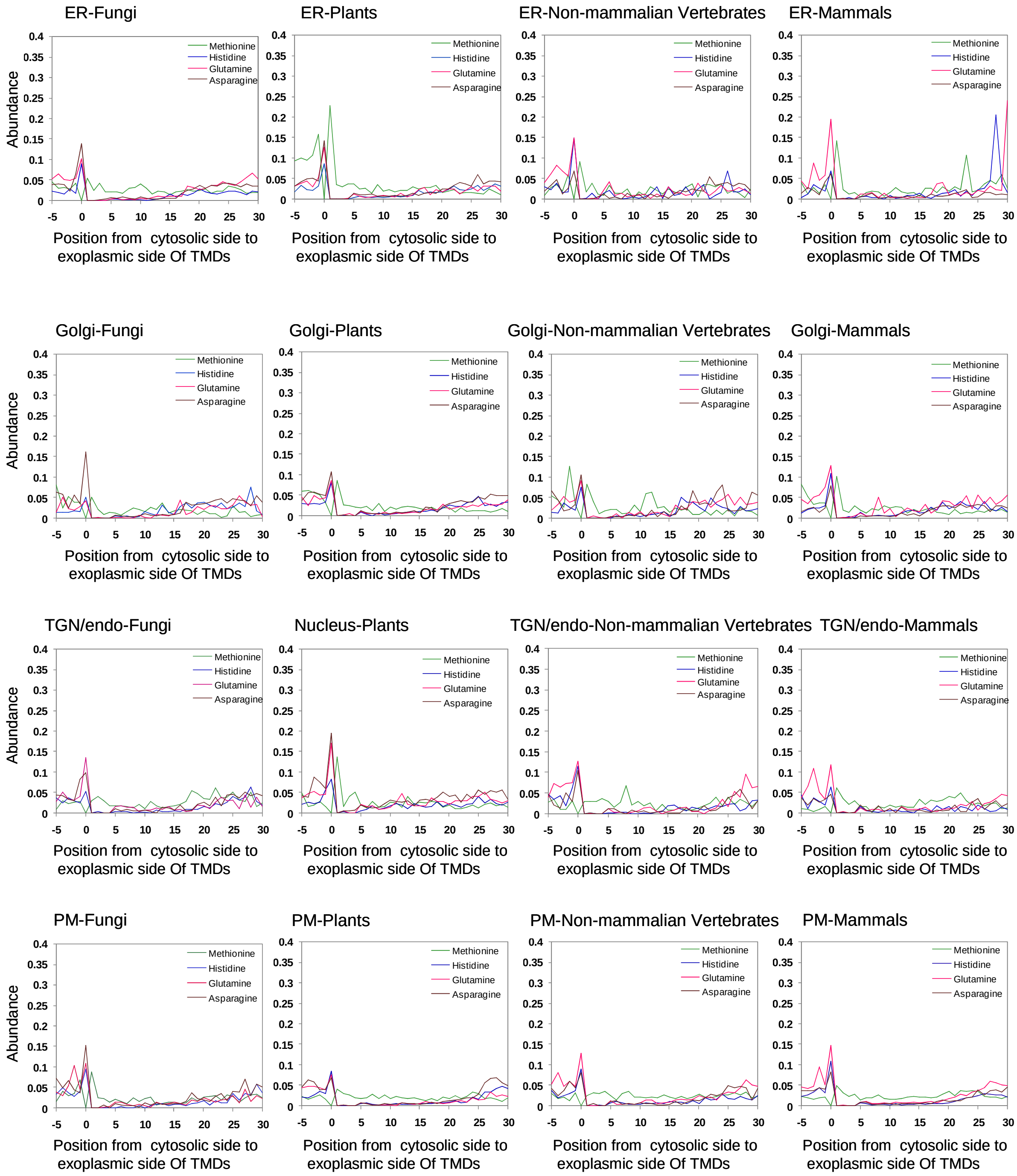

Figure S5: (D) Analysis of the abundance of methionine, histidine, glutamine and asparagine along the TMDs from ER, TGN/endo, Golgi, Nucleus and Plasma membrane in fungi, plants, non-mammalian vertebrates and mammals.

Supplementary Figure S5 (“Explanation” continued on next page)

(E)

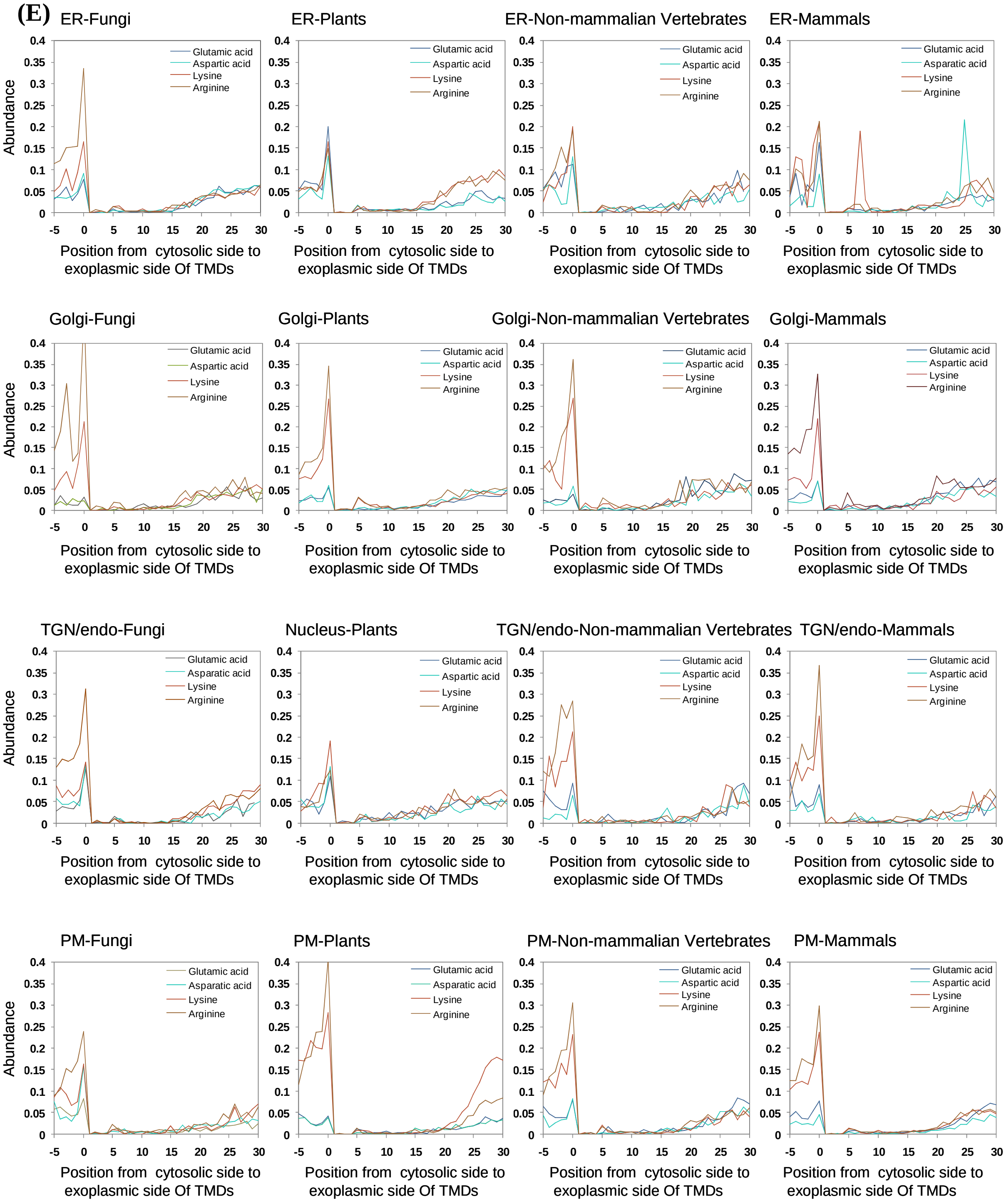

Figure S5: (E) Analysis of the abundance of glutamic acid, aspartic acid, lysine and arginine along the TMDs from ER, TGN/endo, Golgi, Nucleus and Plasma membrane in fungi, plants, non-mammalian vertebrates and mammals.

Supplementary Figure S5 - Explanation

Abundance of amino acids: We have analysed the abundance of all 20 amino acids along the TMDs from all the organelles. Isoleucine and leucine are uniformly distributed through the golgi proteins of fungi, plants, non-mammalian vertebrates and mammals but they are not symmetrically distributed through the plasma membrane in fungi, plants and non-mammalian vertebrates. Valine and glycine are both asymmetrically distributed through the golgi proteins and plasma membrane proteins in all the organelles. Leucine is most abundant in all the organelles in the TMD regions of all the organisms which is clearly seen in the compositional analysis of TMDs (Supplementary Fig. S5A). Phenylalanine was also found most abundant in the core region of TMDs in ER and golgi of fungi and plants (Supplementary Fig. S5B). Prolines were found mostly abundant at the edge of the TMDs rather than in the core (Supplementary Fig. S5C). As expected, polar but somewhat neutral residues i.e. Methionine, Histidine, Glutamine and Asparagine were found sparsely populated in the TMD region in all the organelles (Supplementary Fig. S5D). Further, as expected, charged residues i.e. Glutamic acid, Aspartic acid, Lysine and Arginine are found negligible in the TMD regions but abundant in the non-TMD regions in all the organelles (Supplementary Fig. S5E).

Supplementary Figure S6

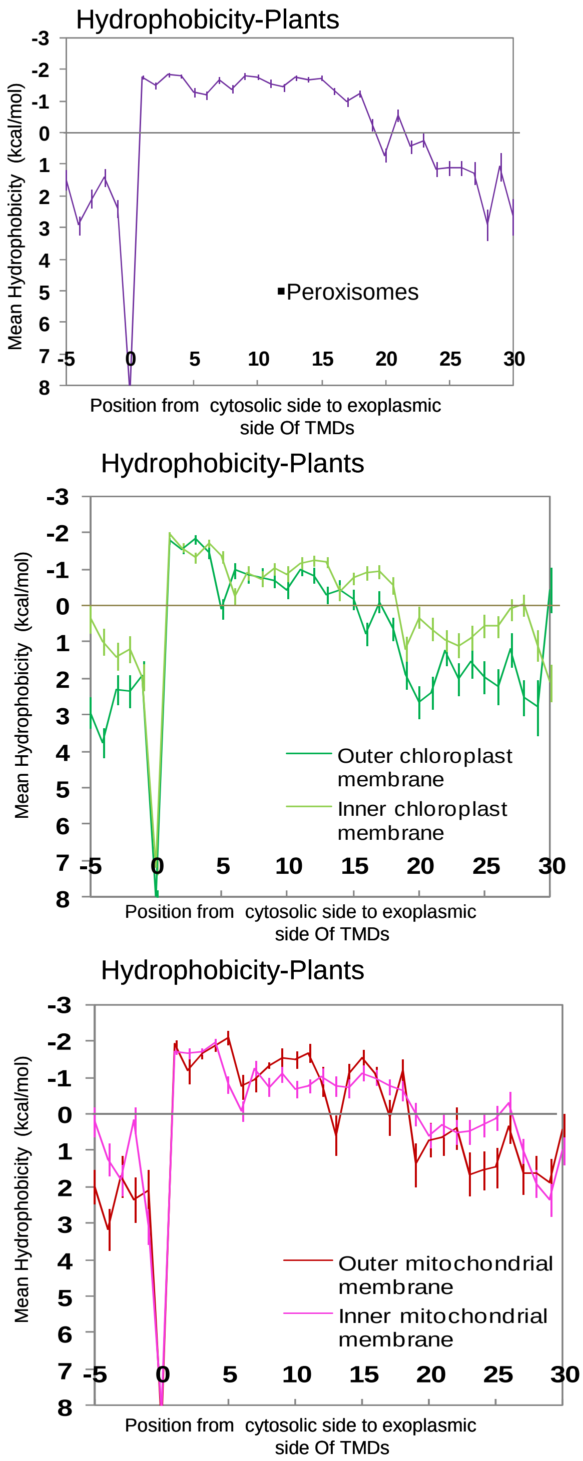

Figure S6: Positional analysis of TMDs of peroxisomes, chloroplast and mitochondria in plants.

Supplementary Figure S7

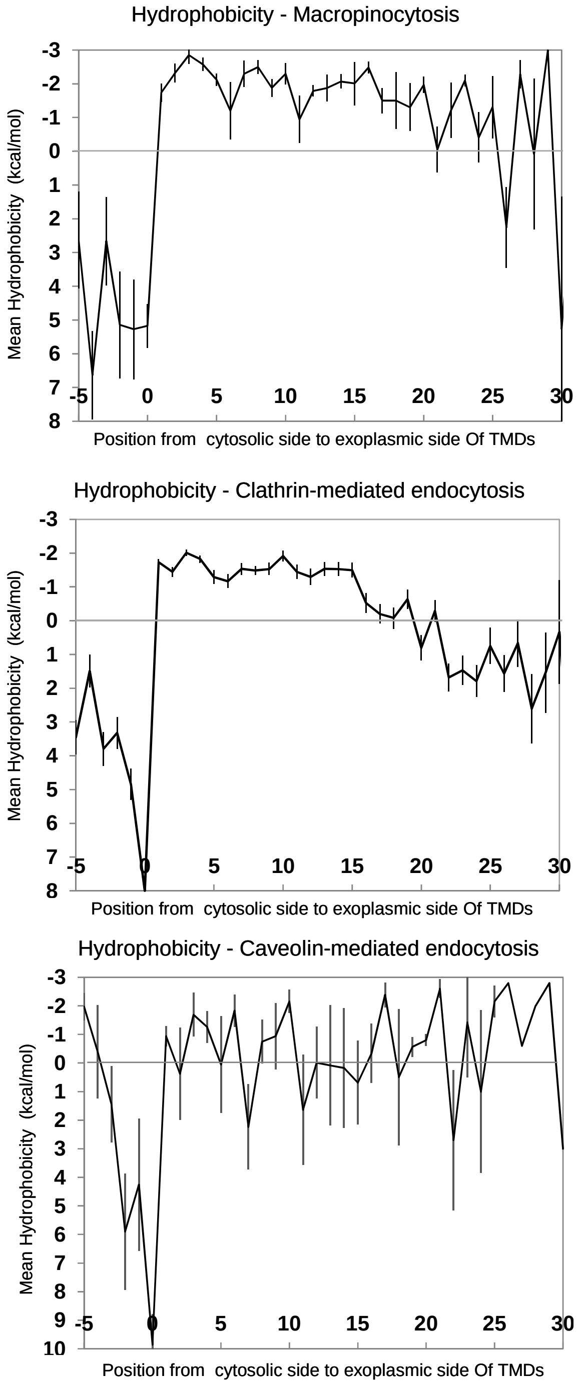

Figure S7: Hydrophobicity graphs of proteins (listed in Table S5, calculations were done after removing redundant sequences as described in methodology) involved in different internalization pathways – macropinocytosis, clathrin-mediated and caveolin-mediated endocytosis.

Supplementary Figure S8

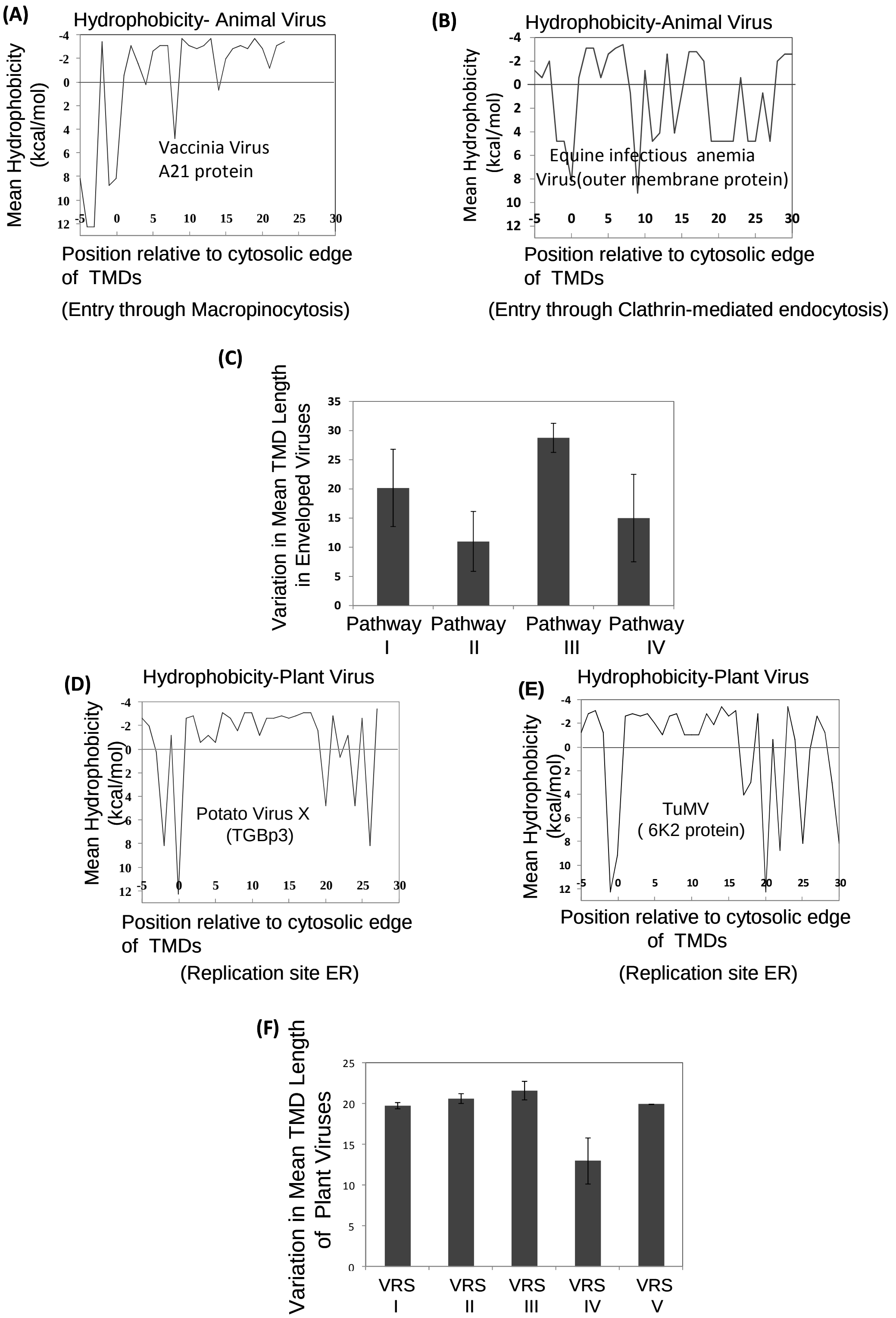

Figure S8: (A) Hydropathy plot - Vaccinia virus (mode of entry into their host cells is through macropinocytosis) **(B)** Hydropathy plot - Equine infectious anemia virus (mode of

entry into their host cells is through clathrin-mediated endocytosis) **(C)** Variation in mean TMD lengths of animal viruses in different entry pathways: Bars show the mean hydrophobic lengths of TMDs (±std) of all the viruses analyzed in this study following different pathways. Viruses entering through clathrin-mediated endocytic pathway into their host cells have shortest TMD lengths where as viruses penetrating through plasma membrane have longest TMD lengths. **(D)** Hydropathy plot of Potato virus X (replication site is endoplasmic reticulum) **(E)** Hydropathy plot of Turnip Mosaic virus (replication site is endoplasmic reticulum) **(F)** Variation in mean TMD lengths of plant viruses in different replication sites: Bars show the mean hydrophobic lengths (±std) of TMDs of different plant viruses in various replication sites. Mean TMD length of those plant viruses is shortest whose replication site is chloroplast where as mean TMD length is longest for those plant viruses whose replication site is peroxisome. However, the data is not populated enough to make any strong conclusions.

Figure S8 clearly indicates that on the basis of TMD lengths and the pattern of hydrophobicity graphs (hydropathy plots) of viral proteomes, one can predict the entry pathways (strongly) and replication sites (quite weakly) for animal and plant viruses respectively.

Supplementary Figure S9

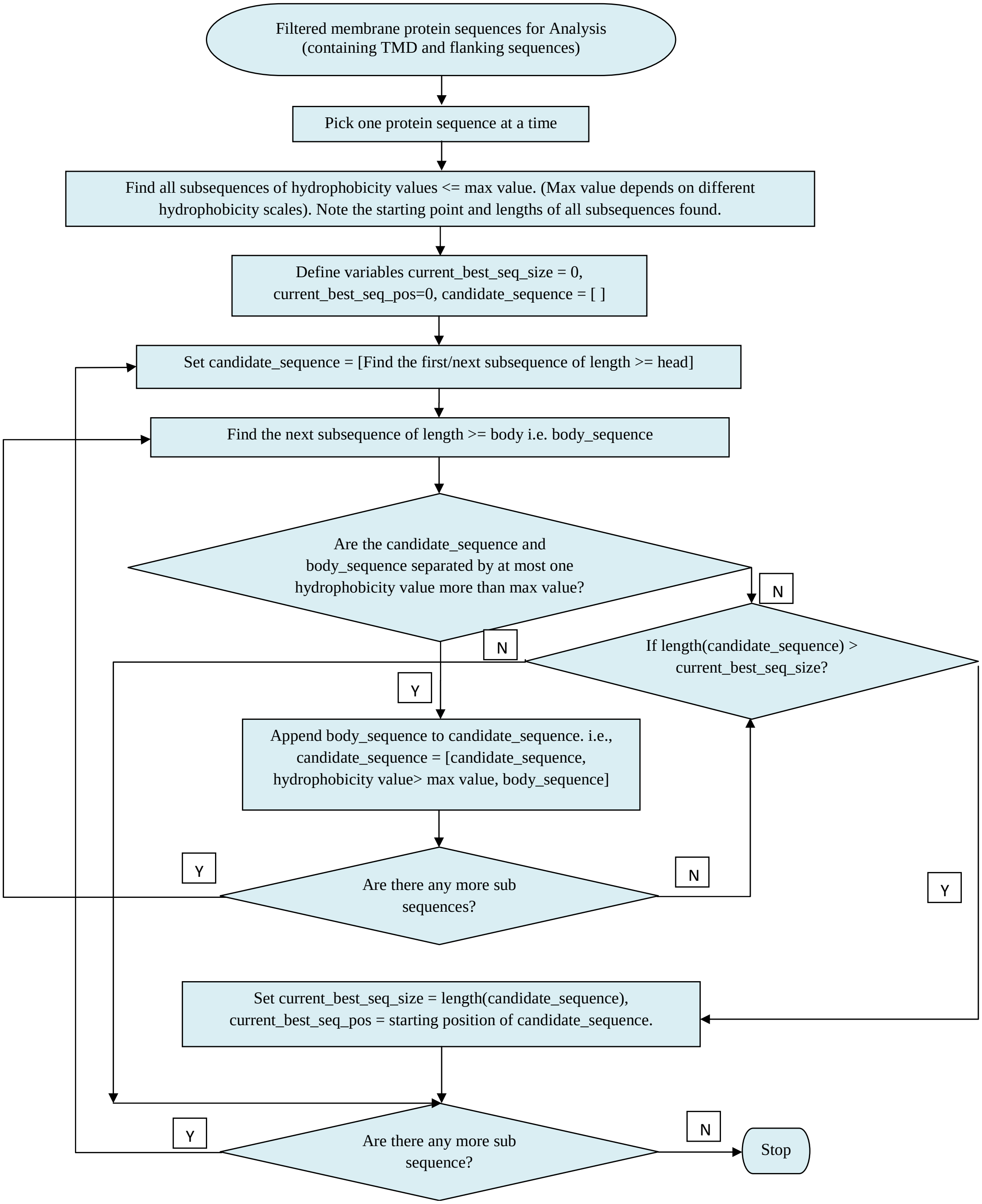

Figure S9: Algorithm for refining edges of TM spans – Implementation was done in MATLAB (Mathworks Inc.). For implementation of the above algorithm, we first define a

“sequence” and a “subsequence” as follows – **Def 1**. A sequence of k numbers is a list of numbers in the form [n1, n2, …, nk], e.g. [1, 2, 3, 1, 1, 0] is a sequence of 6 numbers. **Def 2**. A subsequence (of a sequence) of size r starting from position p is the part of sequence containing r consecutive values starting from the pth position. For example, the sub sequence of size 3 starting from position 4 for example in Def 1 is [1, 1, 0].

When starting a new sub sequence i.e. group of amino acids in a single sequence whose hydrophobicity values <= max value (Max value depends on different hydrophobicity scales and decides the location of amino acids i.e. whether they present in TMDs or outside the TMDs), we first look for a subsequence of size head or more i.e. consecutive amino acid hydrophobicity values that are less than or equal to max value in the beginning of subsequence . Once we find it, we call it the candidate_subsequence. Then we find the next body_sequence of size body or more i.e. number of consecutive values (hydrophobicity values of amino acids that are less than or equal to max value between any two values that are more than max values. If the two are separated by at most one amino acid whose hydrophobicity value > max value, we redefine candidate_subsequence = [candidate_subsequence, one amino acid, body_sequence]. We keep repeating this till the sequence is broken by at least two large entries i.e. two consecutive amino acids whose hydrophobicity values are more than max values, or till we reach the end of whole sequence. Then we check if our candidate subsequence is larger than any other subsequence found earlier i.e. our current group of consecutive amino acids whose hydrophobicity values are less than max value than any other group of amino acids found earlier. If yes, then we replace our “largest so far sequence” by candidate_sequence. Then we check if there are any more subsequences. If yes, we find the next subsequence of size head or more, call it candidate_subsequence and repeat.

Species of fungi from which proteome sequences were collected:

S. cerevisiae , T. phaffii, V. polyspora, T. delbrueckii, Z. rouxii, T. blattae, K. lactis, C. tenuis, D. hansenii, S. passalidarum , M. guilliermondii , S. pombe, C. dubliniensis, B. oryzae , S. stipitis, B. sorokiniana, B. zeicola , W. sebi , C. militaris , L. maculans , S. japonicus y, P. teres , K. pastoris , P. tritici-repentis Pt-1C-BFP, C. lusitaniae, K. pastoris, P. tritici-repentis Pt-1C-BFP, C. lusitaniae ATCC 42720, C. neoformans var. neoformans JEC21, A. capsulatus NAm1, A. niger CBS 513.88, A. dermatitidis SLH14081, P. strigosozonata HHB-11173 SS5, P. chrysogenum Wisconsin 54-1255, A. terreus NIH2624, A. otae CBS 113480, E. lata UCREL1, N. fischeri NRRL 181, C. puteana RWD-64-598 SS2, T. rubrum CBS 118892, Y. lipolytica, M. globosa CBS 7966, C. immitis RS, U. reesii 1704, A. benhamiae CBS 112371, M. brunnea f. sp. 'multigermtubi' MB_m1, C. posadasii C735 delta SOWgp, T. verrucosum HKI 0517, B. dendrobatidis JAM81, A. clavatus NRRL 1, P. flocculosa PF-1, F. graminearum PH-1, T. mesenterica DSM 1558, A. nidulans FGSC A4, L. elongisporus NRRL YB-4239, C. globosum CBS 148.51, T. stipitatus ATCC 10500, P. anserina S mat+, M. anisopliae ARSEF 23, B. dendrobatidis JAM81, C. thermophilum var. thermophilum DSM 1495, S. macrospora k-hell, E. pusillum Z07020, T. terrestris NRRL 8126, G. trabeum ATCC 11539, S. commune H4-8, M. thermophila ATCC 42464, N. parvum UCRNP2, C. albicans SC5314, S. sclerotiorum 1980, C. fioriniae PJ7, M. acridum CQMa 102, T. reesei QM6a, A. flavus NRRL3357, P. carnosa HHB-10118-sp, M. larici-populina 98AG31, C. yegresii CBS 114405, B. compniacensis UAMH 10762, T. versicolor FP-101664 SS1, F. mediterranea MF3/22, S. hirsutum FP-91666 SS1, N. haematococca mpVI 77-13-4, M. roreri MCA 2997, P. graminis f. sp. tritici CRL 75-36-700-3, P. fijiensis CIRAD86, D. squalens LYAD-421 SS1, C. psammophila CBS 110553, P. nodorum SN15, C. epimyces CBS 606.96, P. nodorum SN15, C. epimyces CBS 606.96, C. coronata CBS 617.96, A. delicata TFB-10046 SS5, Z. tritici IPO323, P. fici W106-1, T. minima UCRPA7, G. ATCC 20868, S. lacrymans var.

lacrymans S7.9, C. cinerea okayama7#130, L. bicolor S238N-H82, A. bisporus var. burnettii JB137-S8, A. bisporus var. bisporus H97, M. farinosa CBS 7064, C. glabrata CBS 138, P. carnosa HHB-10118-sp, N. castellii CBS 4309, K. africana CBS 2517, V. polyspora DSM 70294, A. gossypii ATCC 10895, L. thermotolerans, E. cymbalariae DBVPG#7215, N. dairenensis CBS 421, M. farinosa CBS 7064, S. stipitis CBS 6054, M. guilliermondii ATCC 6260, D. hansenii CBS767, S. passalidarum NRRL Y-27907, C. lusitaniae ATCC 42720, Y. lipolytica, U. reesii 1704, T. rubrum CBS 118892, T. verrucosum HKI 0517, A. benhamiae CBS 112371, A. otae CBS 113480, A. gypseum CBS 118893, C. posadasii C735 delta SOWgp, N. parvum UCRNP2, T. stipitatus ATCC 10500, A. delicata TFB-10046 SS5, A. dermatitidis SLH14081, T. terrestris NRRL 8126, E. lata UCREL1, A. fumigatus Af293, A. nidulans FGSC A4, P. nodorum SN15, P. chrysogenum Wisconsin 54-1255, T. melanosporum Mel28, C. globosum CBS 148.51, P. tritici-repentis Pt-1C-BFP, B. sorokiniana ND90Pr, M. oryzae 70-15, S. sclerotiorum 1980, P. flocculosa PF-1, P. fijiensis CIRAD86, T. minima UCRPA7, V. alfalfae VaMs.102, U. maydis 521, S. turcica Et28A, C. thermophilum var. thermophilum DSM 1495, E. pusillum Z07020, G. lozoyensis ATCC 20868, A. capsulatus NAm1, M. acridum CQMa 102, M. brunnea f. sp. 'multigermtubi' MB_m1, Z. tritici IPO323, S. hirsutum FP-91666 SS1, B. compniacensis UAMH 10762, M. anisopliae ARSEF 23, C. militaris CM01, C. gloeosporioides Nara gc5, K. lactis NRRL Y-1140, U. reesii 1704, V. alfalfae VaMs.102, C. puteana RWD-64-598 SS2, D. squalens LYAD-421 SS1.

Supplementary Table S1: Reference proteins from *S.cerevisieae*

**ER**	**Golgi**	**TGN/endo**	**PM**
**NCBI accession**	**NCBI accession**	**NCBI accession**	**NCBI accession**
NP_009343.1	NP_010916.1	NP_014161.1	NP_010898.1
NP_009730.3	NP_015485.1	NP_011312.1	NP_013774.1
NP_009878.1	NP_010531.1	NP_009536.1	NP_011528.3
NP_010618.1	NP_013592.1	NP_015404.1	NP_011537.1
NP_010914.3	NP_014965.3	NP_012095.1	NP_012126.1
NP_010936.1	NP_012352.2	NP_014862.3	NP_013026.2
NP_011011.3	NP_011257.1	NP_013894.1	NP_010708.1
NP_011026.3	NP_011659.3		NP_013185.1
NP_011046.3	NP_012609.3		NP_013436.1
NP_012058.1	NP_012349.2		NP_014116.1
NP_012060.1	NP_011477.1		NP_014536.1
NP_012261.1	NP_012721.1		NP_014650.1
NP_012299.1	NP_009571.1		NP_015400.1
NP_012532.3	NP_012251.3		
NP_012905.1	NP_013465.1		
NP_013308.1	NP_012396.1		
NP_013884.1	NP_009758.3		
NP_009626.1	NP_012987.3		
NP_010371.1	NP_014369.3		
NP_012665.3	NP_015272.1		
NP_014423.1	NP_010771.1		
NP_013167.1	NP_014742.1		
NP_014008.1	NP_009764.3		
NP_014288.3			
NP_014405.1			
NP_015150.2			
NP_011946.1			
NP_012768.1			

Species of plants from which proteome sequences were collected:
A. thaliana, A. lyrata subsp. Lyrata, E. salsugineum, V. vinifera, T. cacao, P. trichocarpa, G. max, R. communis, C. arietinum, P. persica, C. sativus, F. vesca subsp. Vesca, P. vulgaris, C. clementina, C. sinensis, M. domestica, P. mume, S. tuberosum, S. lycopersicum, C. melo, A. trichopoda, O. sativa Japonica Group, S. italic, S. bicolor, B. distachyon, O. brachyantha, C. rubella, T. cacao, Z. mays, M. Truncatula

Supplementary Table S2: Reference proteins from *A.thaliana*

ER	Golgi	Nucleus	PM
**NCBI accession**	**NCBI accession**	**NCBI accession**	**NCBI accession**
NP_565035.1	NP_179585.3	NP_564846.1	NP_188368.2
NP_171714.2	NP_173257.2	NP_566380.2	NP_197162.1
NP_564083.1	NP_179754.1	NP_196118.1	NP_196833.2
NP_173608.1	NP_566611.1	NP_563923.1	NP_177697.1
NP_180150.1	NP_563804.4	NP_564287.1	NP_180241.1
NP_180103.2	NP_683468.1	NP_176892.1	NP_175957.1
NP_178281.1	NP_192086.1	NP_001154799.1	NP_196345.1
NP_188924.3	NP_177220.2	NP_178405.2	NP_190214.1
NP_568917.1	NP_193730.1	NP_566705.1	NP_197789.1
NP_177650.1	NP_192084.1	NP_567929.1	NP_181242.3
NP_177736.1	NP_177220.2	NP_177046.1	NP_001031499.1
NP_177463.1	NP_171983.2	NP_187611.2	NP_179051.1
NP_172041.2	NP_177618.2		NP_175600.2
NP_174119.1	NP_173401.1		NP_172600.1
NP_191525.2	NP_175787.1		NP_175601.2
NP_565249.1	NP_176531.1		NP_172580.2
NP_172854.1	NP_564275.1		NP_175592.2
NP_564672.2	NP_197349.2		NP_190218.1
NP_176675.1	NP_565817.2		NP_181242.3
NP_178590.1	NP_564345.1		NP_179051.1
NP_564905.1	NP_564255.1		NP_175600.2
NP_175469.2	NP_564297.1		NP_175592.2
NP_172633.1	NP_175565.2		NP_190218.1
NP_173458.1	NP_563728.1		NP_190742.1
NP_027545.1	NP_177675.1		NP_569046.1
NP_179363.1	NP_177675.1		NP_172468.3
NP_187425.1	NP_564109.1		NP_198672.1
NP_187425.1	NP_565926.1		NP_190723.1
NP_188924.3	NP_565960.1		NP_180241.1
NP_568202.1	NP_566170.1		NP_180747.1
NP_198817.1	NP_566675.1		
NP_568395.1	NP_564345.1		
NP_197962.1	NP_179627.2		
NP_196868.1	NP_850241.1		
	NP_565485.1		
	NP_187467.1		
	NP_191796.1		
	NP_189024.1		

Species of non-mammalian vertebrates mammals from which proteome sequences were collected:

G. gallus, D. rerio, P. nyererei, M. zebra, A. platyrhynchos, T. guttata, S. salar, F. albicollis, F. peregrines, X. (Silurana) tropicalis, P. humilis, F. cherrug, G. fortis, T. rubripes, X. laevis, O.mykiss, O.latipes, Z. albicollis, C. livia, P. nyrerie, C. picta bellii, M. gallopavo, O. niloticus, F. albicollis, M. Undulatus

Supplementary Table S3: Reference proteins from *G. gallus*

**ER**	**Golgi**	**TGN/endo**	**PM**
**NCBI accession**	**NCBI accession**	**NCBI accession**	**NCBI accession**
NP_001025791.2	NP_001012812.1	XP_424186.2	NP_001019749.1
NP_001026449.1	NP_989759.1	XP_426353.4	NP_001025811.1
NP_001161480.1	NP_001026698.1	XP_003643604.2	XP_424067.4
NP_001006296.1	NP_001012812.1	XP_004938165.1	NP_990189.1
NP_001006561.1	NP_001026647.1	XP_414709.4	XP_004940194.1
NP_990628.1	NP_001072970.1	NP_989958.1	NP_990202.1
NP_001006143.1	NP_989842.1	NP_001004387.1	XP_001234609.3
XP_003643564.1	NP_001001603.1	NP_990500.1	NP_989980.1
XP_004937051.1	NP_001001603.1		NP_001001782.1
XP_001236152.2	NP_001264423.1		NP_989996.1
XP_001236152.2	NP_990548.1		XP_004938384.1
XP_414360.4	NP_990571.1		NP_001180224.1
XP_419082.3	NP_990564.1		XP_003643030.2
XP_416434.4	NP_990572.1		NP_001075887.1
NP_001026316.1	NP_001161219.1		NP_989592.2
NP_001005571.1	NP_001264360.1		NP_990543.1
NP_001026394.1	NP_001026658.1		NP_990709.1
NP_001007947.1	NP_990452.1		XP_417853.3
NP_001029988.2	NP_001025561.1		
XP_430156.3	NP_001026749.1		
XP_422670.2	NP_001012842.1		
XP_415569.4	NP_001026187.1		
NP_001006143.1	NP_001035000.1		
XP_004939048.1	NP_990533.1		
XP_422836.2	NP_990534.1		
	NP_001239028.1		
	NP_001107233.1		
	NP_001004383.1		
	NP_001264768.1		

Species of mammals from which proteome sequences were collected:

H. sapiens , B. acutorostrata scammoni, P. catodon, C. lanigera, S. scrofa, S. harrisii, C. lupus familiaris, M. putorius furo, D. novemcinctus, M. auratus, T. chinensis, P. tigris altaica, P. anubis, T. syrichta, P. catodon, O. anatinus, R. norvegicus, T. truncates, C. hircus, P. maniculatus bairdii, M. musculus, B. bubalis, O. cuniculus, E. telfairi, B. mutus, M. ochrogaster, E. edwardii, E.europaeus, H. glaber, S. araneus, C. porcellus, O. princeps, C. sabaeus, B.taurus, T. chinensis, A. melanoleuca, M. domestica, O. degus, S. harrisii, D. novemcinctus, O. garnettii, J. jaculus, G. gorilla gorilla, N. leucogenys, P. troglodytes, C. jacchus, C. sabaeus, I. tridecemlineatus, S. boliviensis boliviensis, P. tigris altaica, E. caballus, C. cristata, F. catus, D. novemcinctus, M. davidii, T. manatus latirostris, C. lupus familiaris, B. bubalis, P. maniculatus bairdii, B. mutus, O. rosmarus divergens, C. ferus, C. hircus, V. pacos, H. glaber, C. lanigera, P. alecto, P. hodgsonii, M. ochrogaster, P. catodon, C. asiatica, L. vexillifer, O. aries, C. simum simum, P. abelii, S. scrofa, B. acutorostrata scammoni, O. orca, O. princeps, S. araneus, E. fuscus, O. rosmarus divergens, E. europaeus, O. afer afer, M. mulatta, V. pacos, P. tigris altaica, M. putorius furo, C. cristata, L. weddellii.

Supplementary Table S4: Reference proteins from *H.sapiens*

ER	Golgi	TGN/endo	PM
**NCBI accession**	**NCBI accession**	**NCBI accession**	**NCBI accession**
NP_005207.2	NP_005104.3	NP_002950.3	NP_000623.2
NP_057021.2	NP_003587.1	NP_001013049.1	NP_000352.1
NP_005056.3	NP_003586.3	NP_055793.1	NP_001984.1
NP_847894.1	NP_542780.1	NP_000867.2	NP_001992.1
NP_001065.2	NP_036332.2	NP_057340.2	NP_000065.1
NP_061949.3	NP_775811.2	NP_038479.1	NP_000751.1
NP_001066.1	NP_000139.1	XP_006716052.1	NP_724781.1
NP_001064.1	NP_835368.1	NP_061725.1	NP_002987.1
NP_066962.2	NP_000141.1	NP_061725.1	NP_003174.3
NP_002941.1	NP_116053.3	NP_061732.1	NP_000034.1
NP_004353.1	NP_001008978.1	NP_114088.2	NP_000386.1
NP_003892.2	NP_002025.2	NP_958816.1	NP_001241.1

NP_003181.3	NP_003887.3	NP_061721.2	NP_000607.1
NP_659425.1	NP_110392.1		NP_002380.3
NP_003054.1	NP_009141.2		NP_000112.1
NP_060331.3	NP_001482.1		NP_002200.2
NP_004269.1	NP_057675.1		NP_003801.1
NP_067026.3	NP_005898.2		NP_075598.2
XP_004331703.1	NP_001534.1		NP_000132.3
NP_000771.2	NP_004742.1		NP_005182.1
NP_079374.2	NP_004767.1		NP_000867.2
NP_068747.1	NP_001488.2		NP_001772.1
NP_001077.2	NP_009186.1		NP_002429.1
NP_055489.1	NP_004473.2		NP_001450.2
NP_006450.2	NP_004258.2		NP_001056.1
NP_006143.2	NP_006867.1		NP_004098.1
	NP_036346.1		NP_000890.1
	NP_059132.1		NP_000407.1
	NP_002399.1		NP_003255.2
	NP_065112.1		NP_001018074.1
	NP_078917.2		NP_000409.1
	XP_005261665.1		NP_000192.2
	NP_002401.1		NP_001069.1
			NP_000866.1
			NP_000197.1
			NP_000408.1
			NP_001720.1
			NP_001549.2
			NP_006395.2
			NP_000879.2
			NP_001101.1
			NP_003807.1
			NP_001973.2
			NP_002118.1
			NP_002829.3
			NP_006130.1
			NP_001093256.1
			NP_061324.1
			NP_065434.1
			NP_000011.2
			NP_000940.1
			NP_000630.1
			NP_068813.1
			NP_001265515.1
			NP_002990.2
			NP_004320.2
			NP_004451.2

Supplementary Table S5: List of proteins associated with macropinocytosis, clathrin-mediated – and caveolin-mediated – endocytosis

Endocytic Pathway	Protein Name	Accession Number	Species
Macropinocytosis	platelet-derived growth factor receptor betaA	XP_014939043.1	*Acinonyx jubatus*
	integrin beta-1 isoform 1A	NP_002202.2| in	*Homo sapiens*
	integrin beta-1	XP_008154521.1	*Eptesicus fuscus*
	tyrosine-protein kinase receptor UFO isoform X2	XP_003812512.1	*Pan paniscus*
	tyrosine-protein kinase receptor UFO isoform X3	XP_008137790.1	*Eptesicus fuscus*
	tyrosine-protein kinase receptor UFO isoform X1	XP_009192815.1	*Papio anubis*
	tyrosine-protein kinase receptor UFO	XP_012316765.1	*Aotus nancymaae*
	integrin beta-1 isoform X1	XP_007189912.1	*Balaenoptera acutorostrata scammoni*
	platelet-derived growth factor receptor beta isoform X2	XP_013367670.1	*Chinchilla lanigera*
	platelet-derived growth factor receptor beta isoform X2	XP_012360448.1	*Nomascus leucogenys*
	tyrosine-protein kinase receptor UFO	XP_004060847.1	*Gorilla gorilla gorilla*
	tyrosine-protein kinase receptor UFO isoform X1	XP_007650518.1	*Cricetulus griseus*
	LOW QUALITY PROTEIN: integrin beta-1-like	XP_012786696.1	*Ochotona princeps*
	LOW QUALITY PROTEIN: integrin beta-1-like	XP_009243525.1	*Pongo abelii*
	platelet-derived growth factor receptor alpha isoform X2	XP_006714102.1	*Homo sapiens*
	platelet-derived growth factor receptor alpha	XP_004608091.1	*Sorex araneus*
	platelet-derived growth factor receptor beta-like	XP_005661871.2	*Sus scrofa*
	tyrosine-protein kinase receptor UFO	XP_012589186.1	*Condylura cristata*
	platelet-derived growth factor receptor beta-like	XP_006751633.1	*Leptonychotes weddellii*
Clathrin-mediated	low-density lipoprotein receptor-related protein 3 isoform X1	XP_012881751.1	*Dipodomys ordii*
	low-density lipoprotein receptor-related protein 8 isoform X4	XP_008762161.1	*Rattus norvegicus*
	low-density lipoprotein receptor-related protein 8 isoform X5	XP_011993212.1	*Ovis aries musimon*
	low-density lipoprotein receptor-related protein 6	XP_014307184.1	*Myotis lucifugus*
	low-density lipoprotein receptor isoform 1 precursor	NP_000518.1	*Homo sapiens*
	low-density lipoprotein receptor-related protein 1B	XP_013211038.1	*Microtus ochrogaster*
	low-density lipoprotein receptor-related protein 4 isoform X3	XP_011518406.1	*Homo sapiens*
	transferrin receptor protein 2 isoform X4Heterocephalus glab	XP_004840245.1	*Heterocephalus glaber*
	low-density lipoprotein receptor-related protein 5 isoform X1	XP_013369679.1	*Chinchilla lanigera*
	transferrin receptor protein 1Tarsius syrichta]	XP_008062434.1	*Tarsius syrichta*
	low-density lipoprotein receptor-related protein 2	XP_004674925.1	*Condylura cristata*

	low-density lipoprotein receptor class A domain-containing protein 3	XP_012408029.1	*Sarcophilus harrisii*
	low-density lipoprotein receptor-related protein 2	XP_012860404.1	*Echinops telfairi*
	transferrin receptor protein 2 isoform X2	XP_011912650.1	*Cercoce busatys*
	transferrin receptor protein 1 isoform 1	NP_003225.2	*Homo sapiens*
	receptor protein 1	NP_001009312.1	*Felis catus*
	receptor protein 1 isoform 2	NP_001300894.1	*Homo sapiens*
	low-density lipoprotein receptor-related protein 1B-like	XP_008587166.1	*Galeopterus variegatus*
	transferrin receptor protein 2 isoform X5	XP_012666270.1	*Otolemur garnettii*
	MAM and LDL-receptor class A domain-containing protein 1	XP_011225420.1	*Ailuropoda melanoleuca*
	transferrin receptor protein 2	XP_005371498.1	*Microtus ochrogaster*
	low-density lipoprotein receptor-related protein 8 isoform X1	XP_013203988.1	*Microtus ochrogaster*
	low-density lipoprotein receptor-related protein 6-like	XP_007114085.1	*Physeter catodon*
	low-density lipoprotein receptor-related protein 2-like	XP_006060785.1	*Bubalus bubalis*
	low-density lipoprotein receptor isoform X4	XP_006875187.1	*Chrysochloris asiatica*
	transferrin receptor protein 2	XP_003510681.2	*Cricetulus griseus*
	low-density lipoprotein receptor-related protein 1B-like	XP_012505828.1	*Propithecus coquereli*
	LOW QUALITY PROTEIN: transferrin receptor protein 1	XP_008982367.1	*Callithrix jacchus*
	low-density lipoprotein receptor-related protein 4	XP_004627344.1	*Octodon degus*
	low-density lipoprotein receptor-related protein 1B isoform X4	XP_011758124.1	*Macaca nemestrina*
	low-density lipoprotein receptor-related protein 2-like	XP_007174157.1	*Balaenoptera acutorostrata scammoni*
	low-density lipoprotein receptor-related protein 2-like	XP_006753857.1	*Myotis davidii*
	LOW QUALITY PROTEIN: low-density lipoprotein receptor-related protein 5	XP_012612840.1	*Microcebus murinus*
	low-density lipoprotein receptor-related protein 1B-like	XP_005654214.1	*Sus scrofa*
	transferrin receptor protein 2	XP_004399094.1	*Odobenus rosmarus divergens*
	low-density lipoprotein receptor-related protein 1B-like	XP_010362122.1	*Rhinopithecus roxellana*
	low-density lipoprotein receptor-like isoform X1	XP_011236420.1	*Mus musculus*
	low-density lipoprotein receptor isoform X2	XP_012397333.1	*Sarcophilus harrisii*
	lipoprotein receptor isoform 2 precursor	NP_001239587.1	*Mus musculus*
	low-density lipoprotein receptor-related protein 2-like	XP_014641797.1	*Ceratotherium simum simum*
	low-density lipoprotein receptor-related protein 8-like	XP_013207424.1	*Microtus ochrogaster*
	LOW QUALITY PROTEIN: low-density lipoprotein receptor-related protein 5	XP_013831482.1	*Capra hircus*

	low-density lipoprotein receptor-related protein 1B-like	XP_005200458.2	*Bos taurus*
	transferrin receptor protein 1 isoform X2	XP_012496738.1	*Propithecus coquereli*
	transferrin receptor protein 1	XP_004644608.1	*Octodon degus*
	low-density lipoprotein receptor-related protein 4 isoform X2	XP_007497461.1	*Monodelphis domestica*
	low-density lipoprotein receptor isoform X2	XP_007488582.1	*Monodelphis domestica*
	low-density lipoprotein receptor isoform X2	XP_012585162.1	*Condylura cristata*
	low-density lipoprotein receptor-related protein 1B-like	XP_003763886.1	*Sarcophilus harrisii*
	low-density lipoprotein receptor-related protein 10	XP_003474549.2	*Cavia porcellus*
	transferrin receptor protein 1 isoform X1	XP_005639505.1	*Canis lupus familiaris*
	receptor protein 1	NP_001254781.1	*Heterocephalus glaber*
	transferrin receptor protein 1	XP_001364120.1	*Monodelphis domestica*
	transferrin receptor protein 2	XP_001505111.1	*Equus caballus*
	low-density lipoprotein receptor-related protein 4 isoform X2	XP_013002923.1	*Cavia porcellus*
	low-density lipoprotein receptor-related protein 4	XP_001519450.1	*Ornithorhynchus anatinus*
	low-density lipoprotein receptor isoform X1	XP_011526312.1	*Homo sapiens*
	low-density lipoprotein receptor-related protein 2 isoform X4	XP_008542526.1	*Equus przewalskii*
	low-density lipoprotein receptor-related protein 2	XP_011815992.1	*Colobus angolensis palliatus*
	low-density lipoprotein receptor-related protein 1B isoform X6	XP_011237519.1	*Mus musculus*
	low-density lipoprotein receptor-related protein 1B isoform X1	XP_003581884.2	*Bos taurus*
	low-density lipoprotein receptor-related protein 1B-like	XP_006727745.1	*Leptonychotes weddellii*
	low-density lipoprotein receptor-related protein 2-like	XP_004032799.1	*Gorilla gorilla gorilla*
	low-density lipoprotein receptor-related protein 2 isoform X3	XP_011902308.1	*Cercocebus atys*
	low-density lipoprotein receptor-related protein 4 isoform X3	XP_005578074.1	*Macaca fascicularis*
	low-density lipoprotein receptor-related protein 4	XP_005064978.2	*Mesocricetus auratus*
	low-density lipoprotein receptor class A domain-containing protein 3 isoform X1	XP_005661084.2	*Sus scrofa*
	low-density lipoprotein receptor-related protein 4	XP_002763723.2	*Callithrix jacchus*
	low-density lipoprotein receptor-related protein 8-like	XP_007658590.1	*Ornithorhynchus anatinus*
	low-density lipoprotein receptor	XP_012789970.1	*Sorex araneus*
	low-density lipoprotein receptor	XP_011748364.1	*Macaca nemestrina*
	low-density lipoprotein receptor	XP_012381542.1	*Dasypus novemcinctus*
	LOW QUALITY PROTEIN: low-density lipoprotein receptor-related protein 6	XP_009245765.1	*Pongo abelii*

	low-density lipoprotein receptor-related protein 5-like	XP_008252213.1	*Oryctolagus cuniculus*
	low-density lipoprotein receptor-related protein 5-like	XP_014585043.1	*Equus caballus*
	LOW QUALITY PROTEIN: low-density lipoprotein receptor-related protein 4-like	XP_008988734.1	*Callithrix jacchus*
	low-density lipoprotein receptor-related protein 5	XP_014923340.1	*Acinonyx jubatus*
	low-density lipoprotein receptor-related protein 2-like	XP_007664888.1	*Ornithorhynchus anatinus*
	low-density lipoprotein receptor class A domain-containing protein 3-like	XP_007113202.1	*Physeter catodon*
	low-density lipoprotein receptor-related protein 1B-like	XP_006768719.1	*Myotis davidii*
	low-density lipoprotein receptor-related protein 2-like	XP_007489534.1	*Monodelphis domestica*
	low-density lipoprotein receptor-related protein 1B isoform X3	XP_007652067.1	*Cricetulus griseus*
	LOW QUALITY PROTEIN: low-density lipoprotein receptor class A domain-containing protein 3	XP_014448558.1	*Tupaia chinensis*
	low-density lipoprotein receptor-related protein 3 isoform X1	XP_005653323.1	*Sus scrofa*
	low-density lipoprotein receptor-related protein 8-like	XP_005001140.1	*Cavia porcellus*
	low-density lipoprotein receptor	XP_010327865.1	*Saimiri boliviensis boliviensis*
	low-density lipoprotein receptor	XP_012364768.1	*Nomascus leucogenys*
	low-density lipoprotein receptor	XP_012889401.1	*Dipodomys ordii*
	MAM and LDL-receptor class A domain-containing protein 1	XP_014394873.1	*Myotis brandtii*
	low-density lipoprotein receptor class A domain-containing protein 3	XP_014393681.1	*Myotis brandtii*
	LOW QUALITY PROTEIN: MAM and LDL-receptor class A domain-containing protein 1	XP_013001437.1	*Cavia porcellus*
	low-density lipoprotein receptor	XP_010372129.1	*Rhinopithecus roxellana*
	transferrin receptor protein 1	XP_006884261.1	*Elephantulus edwardii*
	transferrin receptor protein 1	XP_012662345.1	*Otolemur garnettii*
	transferrin receptor protein 2	XP_001371634.2	*Monodelphis domestica*
	LOW QUALITY PROTEIN: transferrin receptor protein 2	XP_014941216.1	*Acinonyx jubatus*
	transferrin receptor protein 2	XP_014709450.1	*Equus asinus*
	LOW QUALITY PROTEIN: transferrin receptor protein 2	XP_008696730.1	*Ursus maritimus*
	transferrin receptor protein 1-like	XP_003585042.2	*Bos taurus*
	low-density lipoprotein receptor-related protein 8	XP_014310147.1	*Myotis lucifugus*
	low-density lipoprotein receptor-related protein 8 isoform X8	XP_011540398.1	*Homo sapiens*
	low-density lipoprotein receptor-related protein 8	XP_006768452.1	*Myotis davidii*
	low-density lipoprotein receptor	XP_009191821.1	*Papio anubis*

	low-density lipoprotein receptor-related protein 4	XP_006984456.1	*Peromyscusmaniculatus bairdii*
	low-density lipoprotein receptor-related protein 4 isoform X1	XP_006499306.1	*Mus musculus*
	low-density lipoprotein receptor-related protein 4	XP_007519216.1	*Erinaceuseuropaeus*
	LOW QUALITY PROTEIN: low-density lipoprotein receptor-related protein 8	XP_012807363.1	*Jaculus jaculus*
	low-density lipoprotein receptor-related protein 2 precursor	NP_110454.1	*Rattus norvegicus*
	LOW QUALITY PROTEIN: low-density lipoprotein receptor	XP_006905916.1	*Pteropus alecto*
	low-density lipoprotein receptor-related protein 2 isoform X2	XP_007669389.1	*Ornithorhynchus anatinus*
	LOW QUALITY PROTEIN: low-density lipoprotein receptor-related protein 2	XP_007494986.1	*Monodelphis domestica*
	low-density lipoprotein receptor-related protein 2	XP_013837666.1	*Sus scrofa*
	LOW QUALITY PROTEIN: low density lipoprotein receptor-related protein 1	XP_007508013.1	*Monodelphis domestica*
	low-density lipoprotein receptor-related protein 2	XP_006902182.1	*Elephantulus edwardii*
	low-density lipoprotein receptor-related protein 2	XP_005346576.1	*Microtus ochrogaster*
	low-density lipoprotein receptor-related protein 2	XP_008256929.1	*Oryctolagus cuniculus*
	low-density lipoprotein receptor-related protein 1B	XP_012968045.1	*Mesocricetus auratus*
	low-density lipoprotein receptor-related protein 2-like	XP_012381523.1	*Dasypus novemcinctus*
	low-density lipoprotein receptor-related protein 1B	XP_004473967.1	*Dasypus novemcinctus*
	low-density lipoprotein receptor-related protein 1B	XP_004375173.1	*Trichechus manatus latirostris*
	LOW QUALITY PROTEIN: low-density lipoprotein receptor-related protein 2	XP_012381521.1	*Dasypus novemcinctus*
	low-density lipoprotein receptor-like	XP_008562823.1	*Galeopterus variegatus*
	low-density lipoprotein receptor-like	XP_008709151.1	*Ursus maritimus*
	low-density lipoprotein receptor-related protein 1-like	XP_013376941.1	*Chinchilla lanigera*
	low-density lipoprotein receptor-related protein 1-like	XP_014309132.1	*Myotis lucifugus*
	low-density lipoprotein receptor-related protein 1B	NP_001101313.1	*Rattus norvegicus*
	low-density lipoprotein receptor-related protein 1B-like	XP_004867723.1	*Heterocephalus glaber*
	low-density lipoprotein receptor-related protein 1-like	XP_005000327.1	*Cavia porcellus*
	low-density lipoprotein receptor-related protein 2-like	XP_012317768.1	*Aotusnancymaae*
	low-density lipoprotein receptor-related protein 1B-like	XP_004321464.1	*Tursiops truncatus*

	low-density lipoprotein receptor-like	XP_007539201.1	*Erinaceus europaeus*
	low-density lipoprotein receptor-related protein 1 isoform X5	XP_014393380.1	*Myotis brandtii*
	LOW QUALITY PROTEIN: low-density lipoprotein receptor	XP_011378606.1	*Pteropus vampyrus*
	low-density lipoprotein receptor-related protein 1B-like	XP_009180405.1	*Papio anubis*
	low-density lipoprotein receptor-related protein 1B-like	XP_008589038.1	*Galeopterus variegatus*
	low-density lipoprotein receptor-related protein 2-like	XP_011225837.1	*Ailuropoda melanoleuca*
	low-density lipoprotein receptor-related protein 1B-like	XP_002812501.1	*Pongo abelii*
	low-density lipoprotein receptor-related protein 1B-like	XP_012505827.1	*Propithecus coquereli*
	low-density lipoprotein receptor-related protein 4-like	XP_011371513.1	*Pteropus vampyrus*
	low-density lipoprotein receptor-related protein 5-like protein	XP_008567790.1	*Galeopterus variegatus*
	low-density lipoprotein receptor precursor	NP_786938.1	*Rattus norvegicus*
	low-density lipoprotein receptor-related protein 5	XP_001374330.1	*Monodelphis domestica*
	low-density lipoprotein receptor-related protein 5	XP_006742753.1	*Leptonychotes weddellii*
	low-density lipoprotein receptor-related protein 5	XP_012423423.1	*Odobenus rosmarus divergens*
	low-density lipoprotein receptor-related protein 5	XP_004618628.1	*Sorex araneus*
	low-density lipoprotein receptor	XP_007526109.1	*Erinaceus europaeus*
	low-density lipoprotein receptor-related protein 5	XP_013976412.1	*Canis lupus familiaris*
	low-density lipoprotein receptor-related protein 5	XP_005578910.1	*Macaca fascicularis*
	LOW QUALITY PROTEIN: transferrin receptor protein 2	XP_003422538.2	*Loxodonta africana*
	LOW QUALITY PROTEIN: low-density lipoprotein receptor-related protein 5-like protein	XP_014717111.1	*Equus asinus*
	low-density lipoprotein receptor	XP_007994273.1	*Chlorocebus sabaeus*
	low-density lipoprotein receptor-related protein 6-like	XP_011216123.1	*Ailuropoda melanoleuca*
	transferrin receptor protein 1	XP_011816639.1	*Colobus angolensis palliatus*
	LOW QUALITY PROTEIN: low-density lipoprotein receptor	XP_007079407.1	*Panthera tigris altaica*
	low-density lipoprotein receptor-related protein 2 isoform X3	XP_011509487.1	*Homo sapiens*
	low-density lipoprotein receptor-related protein 2-like	XP_010620608.1	*Fukomys damarensis*
	low-density lipoprotein receptor-related protein 4-like isoform X2	XP_014962409.1	*Ovis aries musimon*
	low-density lipoprotein receptor-related protein 2-like	XP_008528431.1	*Equus przewalskii*
	low-density lipoprotein receptor-like isoform X3	XP_011236422.1	*Mus musculus*

	low-density lipoprotein receptor-related protein 1B-like	XP_003483638.1	*Sus scrofa*
	low-density lipoprotein receptor-related protein 3	XP_005616955.1	*Canis lupus familiaris*
	low-density lipoprotein receptor-related protein 2-like	XP_008523411.1	*Equus przewalskii*
	low-density lipoprotein receptor-related protein 2 isoform X2	XP_010824740.1	*Bos taurus*
	low-density lipoprotein receptor-related protein 1B-like	XP_003793073.1	*Otolemur garnettii*
	low-density lipoprotein receptor-like	XP_004321103.1	*Tursiops truncatus*
	low-density lipoprotein receptor-related protein 1B-like	XP_014587742.1	*Equus caballus*
	LOW QUALITY PROTEIN: low-density lipoprotein receptor-related protein 1B-like	XP_009180408.1	*Papio anubis*
	low-density lipoprotein receptor-related protein 4-like isoform X1	XP_012966161.1	*Mesocricetus auratus*
	low-density lipoprotein receptor-related protein 6-like	XP_007076389.1	*Panthera tigris altaica*
	LOW QUALITY PROTEIN: low-density lipoprotein receptor-related protein 5-like protein	XP_014440794.1	*Tupaia chinensis*
	low-density lipoprotein receptor-related protein 1B-like	XP_013837390.1	*Sus scrofa*
	low-density lipoprotein receptor-related protein 2-like	XP_006040727.1	*Bubalus bubalis*
	low-density lipoprotein receptor-related protein 10	XP_537364.2	*Canis lupus familiaris*
	low-density lipoprotein receptor-related protein 8	XP_006737735.1	*Leptonychotes weddellii*
	low-density lipoprotein receptor-related protein 1B-like	XP_007109711.1	*Physeter catodon*
	low-density lipoprotein receptor-like	XP_003945116.2	*Saimiri boliviensis boliviensis*
Caveolin-mediated	caveolin-2 isoform c	NP_937855.1	*Homo sapiens*
	PREDICTED: caveolin-2 isoform X2	XP_007980865.1	*Chlorocebus sabaeus*
	PREDICTED: caveolin-2 isoform X1	XP_005205450.1	*Bos taurus*
	PREDICTED: caveolin-2 isoform X2	XP_005959438.1	*Pantholops hodgsonii*
	PREDICTED: hypothetical protein LOC100655716	XP_003407275.1	*Loxodonta africana*
	PREDICTED: caveolin-2 isoform X2	XP_008534172.1	*Equus przewalskii*
	PREDICTED: caveolin-2 isoform X1	XP_005628405.1	*Canis lupus familiaris*
	caveolin-2	NP_001162006.1	*Pongo abelii*
	PREDICTED: caveolin-2-like isoform X3	XP_005080993.1	*Mesocricetus auratus*
